# Blood RNA biomarkers in prodromal PARK4 and rapid eye movement sleep behavior disorder show role of complexin 1 loss for risk of Parkinson's disease

**DOI:** 10.1242/dmm.028035

**Published:** 2017-05-01

**Authors:** Suna Lahut, Suzana Gispert, Özgür Ömür, Candan Depboylu, Kay Seidel, Jorge Antolio Domínguez-Bautista, Nadine Brehm, Hülya Tireli, Karl Hackmann, Caroline Pirkevi, Barbara Leube, Vincent Ries, Kerstin Reim, Nils Brose, Wilfred F. den Dunnen, Madrid Johnson, Zsuzsanna Wolf, Marc Schindewolf, Wiebke Schrempf, Kathrin Reetz, Peter Young, David Vadasz, Achilleas S. Frangakis, Evelin Schröck, Helmuth Steinmetz, Marina Jendrach, Udo Rüb, Ayşe Nazlı Başak, Wolfgang Oertel, Georg Auburger

**Affiliations:** 1 Experimental Neurology, Goethe University Medical School, Frankfurt/Main 60590, Germany; 2 NDAL, Boğaziçi University, Istanbul 34342, Turkey; 3 Department of Neurology, Philipps University, Baldingerstrasse, Marburg 35043, Germany; 4 Dr Senckenberg Chronomedical Institute, Goethe University, Frankfurt/Main 60590, Germany; 5 Department of Neurology, Haydarpaşa Numune Training and Research Hospital, Istanbul 34668, Turkey; 6 Institute for Clinical Genetics, Faculty of Medicine Carl Gustav Carus, TU Dresden, Fetscherstrasse 74, Dresden 01307, Germany; 7 Institute of Human Genetics, Heinrich Heine University, Düsseldorf 40225, Germany; 8 Department of Molecular Neurobiology andCenter for the Molecular Physiology of the Brain, Max Planck Institute of Experimental Medicine, Göttingen 37075, Germany; 9 Department of Pathology and Medical Biology, Medical Center, University, Groningen 9700 RB, The Netherlands; 10 Buchmann Institute for Molecular Life Sciences and Institute for Biophysics, Goethe University, Frankfurt/Main 60438, Germany; 11 Haemophilia Centre, Medical Clinic III, Institute of Immunohaematology and Transfusion Medicine, Goethe University, Frankfurt/Main 60590, Germany; 12 Department of Internal Medicine, Division of Vascular Medicine and Hemostaseology, Goethe University, Frankfurt 60590, Germany; 13 Division of Neurodegenerative Diseases, Department of Neurology, Technische Universität, Dresden 01307, Germany; 14 Department of Neurology, RWTH Aachen University Hospital, Aachen 52074, Germany; 15 Department of Sleep Medicine and Neuromuscular Disorders, University Hospital Münster, Münster 48149, Germany

**Keywords:** α-synuclein, Complexin 1, PARK4, Rapid eye movement sleep behavior disorder, Parkinson's disease, Biomarkers

## Abstract

Parkinson's disease (PD) is a frequent neurodegenerative process in old age. Accumulation and aggregation of the lipid-binding SNARE complex component α-synuclein (SNCA) underlies this vulnerability and defines stages of disease progression. Determinants of SNCA levels and mechanisms of SNCA neurotoxicity have been intensely investigated. In view of the physiological roles of SNCA in blood to modulate vesicle release, we studied blood samples from a new large pedigree with *SNCA* gene duplication (PARK4 mutation) to identify effects of SNCA gain of function as potential disease biomarkers. Downregulation of complexin 1 (*CPLX1*) mRNA was correlated with genotype, but the expression of other Parkinson's disease genes was not. In global RNA-seq profiling of blood from presymptomatic PARK4 indviduals, bioinformatics detected significant upregulations for platelet activation, hemostasis, lipoproteins, endocytosis, lysosome, cytokine, Toll-like receptor signaling and extracellular pathways. In PARK4 platelets, stimulus-triggered degranulation was impaired. Strong *SPP1*, *GZMH* and *PLTP* mRNA upregulations were validated in PARK4. When analysing individuals with rapid eye movement sleep behavior disorder, the most specific known prodromal stage of general PD, only blood *CPLX1* levels were altered. Validation experiments confirmed an inverse mutual regulation of *SNCA* and *CPLX1* mRNA levels. In the 3′-UTR of the *CPLX1* gene we identified a single nucleotide polymorphism that is significantly associated with PD risk. In summary, our data define *CPLX1* as a PD risk factor and provide functional insights into the role and regulation of blood SNCA levels. The new blood biomarkers of PARK4 in this Turkish family might become useful for PD prediction.

## Abstract (precis)

**Summary:** Complexin 1 is a prodromal biomarker and risk factor for REM sleep behavior disorder and PARK4-associated Parkinson's disease.

## INTRODUCTION

Parkinson's disease (PD) is the second most frequent age-associated brain degeneration disorder, affecting ∼1% of the population >65 years of age. The PD-specific progressive movement deficit is mostly attributable to the severe affliction and cell death of midbrain nigrostriatal dopaminergic neurons (Braak et al., 2003). Surviving neurons in vulnerable regions exhibit aggregates predominantly consisting of the protein α-synuclein (SNCA), which are visualized as Lewy neurites and Lewy bodies, both in sporadic late-onset PD and in most familial early-onset PD variants (Goedert et al., 2013).

Autosomal dominant PD with early clinical manifestation was observed in rare families, leading to the identification of SNCA protein missense mutations (PARK1 variant), such as A53T, and of *SNCA* gene duplication/triplication events (PARK4 variant) as the strongest causes of this pathology (Polymeropoulos et al., 1997; Singleton et al., 2003). Further recruitment of PD families led to the identification of several disease genes responsible for monogenic PD (Corti et al., 2011). In addition, genome-wide association studies (GWAS) of very large collectives of late-manifesting sporadic PD cases identified two regions on chromosome 4 (*SNCA* locus and *CPLX1/GAK/TMEM175*/*DGKQ* locus) that contain genetic variants predisposing to multifactorial PD (Lill et al., 2012; Nalls et al., 2014). Variations in the *SNCA* gene 3′-untranslated region (3′-UTR) and its promoter were strongly correlated with PD risk (Rhinn et al., 2012).

SNCA is physiologically concentrated in axon terminals. It is associated with the lipid membranes of synaptic vesicles and interacts with synaptobrevin, a component of the SNARE complex, modulating vesicle exocytosis and neurotransmission (Diao et al., 2013). Its toxic gain of function leads over time to impaired synaptic vesicle release and synaptic failure (Garcia-Reitbock et al., 2010; Nemani et al., 2010; Platt et al., 2012; Janezic et al., 2013). Current investigations aim to elucidate SNCA-triggered pathology, concentrating on disease stages before the occurrence of irreversible cell loss, when neuroprotective therapies might still be efficacious. In the prodromal stage of PD, non-motor symptoms such as hyposmia, constipation, depression or rapid eye movement (REM) sleep behavior disorder (RBD) were documented, of which RBD is now recognized as the most specific and predictive prodromal phenotype. Individuals suffering from RBD carry a risk of >85% to manifest PD after 15-20 years, and the associated neurodegenerative process is a synucleinopathy in 95% of cases (Stiasny-Kolster et al., 2005; Albers et al., 2012; Boeve et al., 2013; Iranzo et al., 2013, 2014; Mahowald and Schenck, 2013).

SNCA is abundantly expressed in blood (Shin et al., 2000; Barbour et al., 2008). The accumulation of SNCA in short-lived blood cells was found to result in diverse subtle phenotypes. Enhanced apoptotic vulnerability of human PARK1 lymphocytes and SNCA-transfected myeloma and leukemia cell lines to oxidative stress (Kim et al., 2004; Battisti et al., 2008), impaired innate immune functions of mouse leukocytes with SNCA overexpression (Gardai et al., 2013) and dose-dependent inhibition of α-granule release in human platelets exposed to exogenous SNCA (Park et al., 2002) provide evidence that biomarkers of elevated SNCA abundance and of the risk of synucleinopathy can be identified in peripheral tissues.

Our identification of a new large pedigree of autosomal dominant PD attributable to *SNCA* gene duplication with 12 presymptomatic PARK4 heterozygotes has provided a unique opportunity to explore blood biomarkers and permitted the definition of a molecular signature at the RNA level that predicts PARK4 PD. For validation, the results were assessed in individuals with a risk of developing PD because of manifestation of RBD as a highly specific prodromal sign. Our data on blood biomarkers as a diagnostic tool might contribute to the assessment of the risk of multifactorial PD in individuals without a positive family history. The most relevant biomarker is the SNARE component complexin 1, which acts as risk factor for PD by itself.

## RESULTS

### RNA levels in blood from presymptomatic PARK4 heterozygotes are reduced for *CPLX1*

**Fig. 1.**
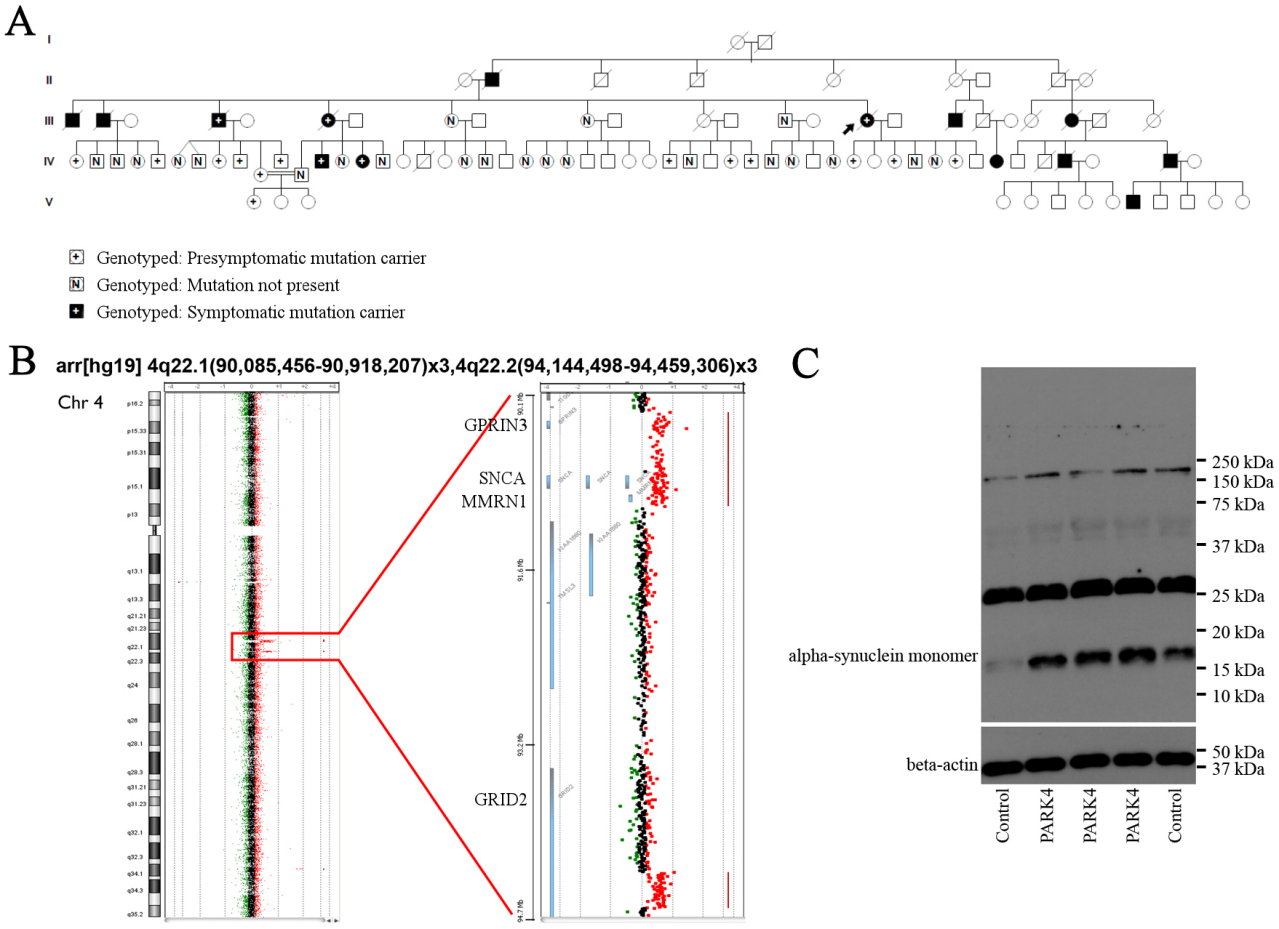
**Large kindred with autosomal dominant PD inheritance attributable to PARK4 mutation with 12 presymptomatic PARK4 heterozygotes.** (A) Pedigree structure with genotype of *SNCA* gene duplication. Squares and circles denote male and female individuals, respectively. Black filling versus the black symbol with ‘+’ inside illustrates clinically manifested PD versus presymptomatic PARK4 heterozygote status, respectively. The letter ‘N’ indicates the individuals genotyped and found not to carry the PARK4 mutation. Samples from the rest of the individuals were unavailable to us; therefore, their status was indicated according to the information gained from the family. (B) DNA from the Turkish PARK4 family exhibits duplications at the *SNCA* locus. The analysis of the chromosome 4q22.1-22.2 region by molecular karyotyping using Agilent array CGH revealed two adjacent copy number gains on chromosome 4 (left chromosome view, right gene view). The first duplication, 833-862 kb in size, carries *GPRIN3*, *SNCA* and *MMRN1* in their entirety. The second duplication, in close proximity, affects exons 7-13 of the *GRID2* gene. The genomic position coordinates as annotated by hg19 nomenclature are indicated above for both chromosome 4 fragments that exist in three copies. (C) PARK4 blood shows upregulation of α-synuclein monomer, but no high molecular weight aggregates in the limited mobility zone of gels. Whole blood protein extracts were depleted in hemoglobin, analyzed on polyacrylamide blots subjected to delipidation, then studied for α-synuclein (above) and β-actin (below, as a loading control) immunoreactivity.

**Fig. 2.**
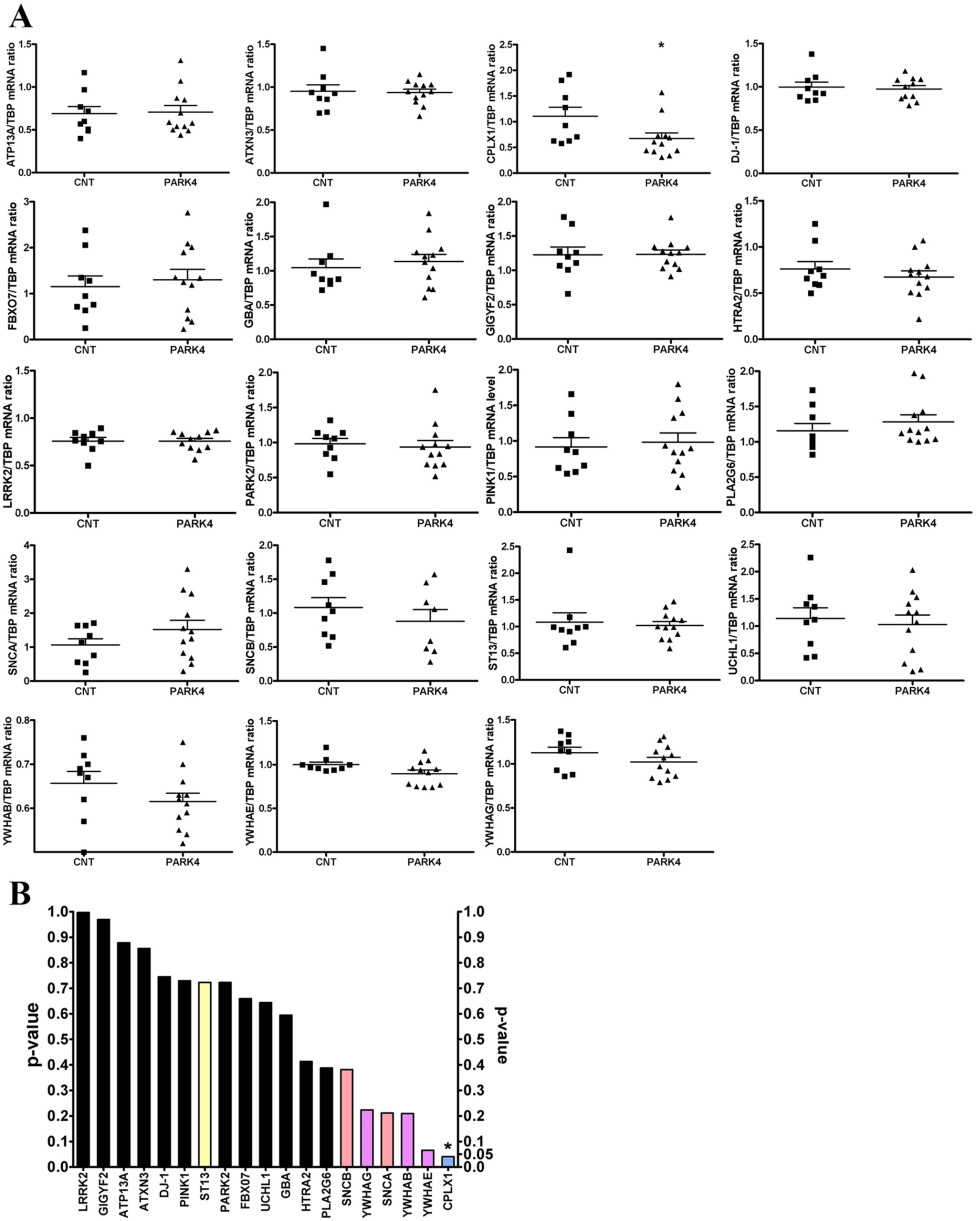
**Correlation between whole blood mRNA levels (qPCR) of candidate genes and PARK4 genotype, comparing 14 adult PARK4 heterozygotes versus nine age-matched control relatives.** (A) Scattergrams represent the expression levels in whole blood of individual transcripts determined by qPCR and normalized versus TBP loading control, with mean and s.e.m. (B) The bar graph summarizes these scattergrams by illustrating nominal Student's unpaired *t*-test *P*-values. The selected candidate genes are either known to be mutated in monogenic PD (black) or were previously claimed to constitute a blood expression biomarker of PD (yellow) or represent components of the interactome of α-synuclein (SNCA), including β-synuclein (SNCB) (orange), 14-3-3 isoforms (YWHAG, YWHAB and YWHAE) (purple) and complexin 1 (CPLX1) (blue) within the presynaptic SNARE complex. *P<0.05.

An exceptionally large pedigree with autosomal dominant PD (Fig. 1A) was identified in Turkey. Blood DNA genotyping demonstrated a genomic tandem duplication at the *SNCA* locus as a known cause of PD (Singleton et al., 2003; Fig. 1B) and detected presymptomatic PARK4 heterozygotes, 12 of whom were available and included in this study. The two clinically affected family members plus the 12 presymptomatic heterozygotes (mean age 45.5 years, range 29-56 years, six males) and the 12 age-matched control relatives (mean age 44.6 years, range 31-57 years, six males) underwent overnight fasting and had whole peripheral blood protein and RNA samples collected and processed in parallel to assess the SNCA-dependent expression profiles of blood. As a consequence of the tandem duplication, the blood mRNA levels of the neighbouring genes *GPRIN3*, *SNCA* and *MMRN1* were increased to ∼1.5-fold in blood of PARK4 individuals versus control relatives (11 versus 9). Given that the *MMRN1* gene dosage has also been implicated in cognitive decline (Nishioka et al., 2006; Fuchs et al., 2007; Mutez et al., 2011), it will be interesting to perform a neuropsychological characterization of the PARK4 heterozygotes in this family in the future, but currently the cognitive score in one patient is still unaffected (mini-mental state examination score, MMSE=30). In an initial candidate gene study, we studied the blood expression of all PARK genes, of the previously reported PD blood biomarker *ST13* and of promising transcripts that encode putative SNCA-interactor proteins (Fig. 2A,B) and showed altered levels in our previous global transcriptome profiling of midbrain tissue in a carefully characterized synucleinopathy mouse model with dopaminergic deficits, impaired synaptic plasticity and mitochondrial dysfunction (Kurz et al., 2010; Platt et al., 2012; Tozzi et al., 2012; Gispert et al., 2015a,b; Subramaniam et al., 2014; Brehm et al., 2015b). *SNCA* gene duplication in the Turkish pedigree showed no correlation with the expression of other genes with known association with PD risk or of the previously reported PD blood biomarker *ST13* (Scherzer et al., 2007). A nominally significant inverse correlation was observed for *CPLX1* and a trend towards inverse correlation for *YWHAE* (Fig. 2B), two phenomena that we had previously identified as synucleinopathy markers in mouse mutant midbrain (Gispert et al., 2015b; Brehm et al., 2015a). *CPLX1* levels were reduced to 0.67-fold, so the effect size was moderate; despite the nominal significance (*P*=0.04), a demonstration of real significance after Bonferroni or Benjamini–Hochberg correction would be possible only in much larger sample collections. Quantitative immunoblots of protein extracts from corresponding whole blood PARK4 samples showed a ∼1.5-fold accumulation of SNCA monomer, but no SNCA aggregates were detectable, even after delipidation for epitope unmasking, probably because of the short lifespan of blood cells (Sharon et al., 2003; Fig. 1C). The protein levels of complexin 1 could not be detected reliably in blood. Together with previous mouse findings (Chandra et al., 2004; Gispert et al., 2015b), these data suggest that *CPLX1* mRNA levels in blood mirror the gain of physiological function of SNCA rather than aggregation pathology. This provides proof of principle that blood RNA profiling can aid the development of risk prediction biomarkers for synucleinopathies.

### Blood platelets from presymptomatic PARK4 heterozygotes suggest reduced stimulus-triggered degranulation in the absence of SNCA aggregates

**Fig. 3.**
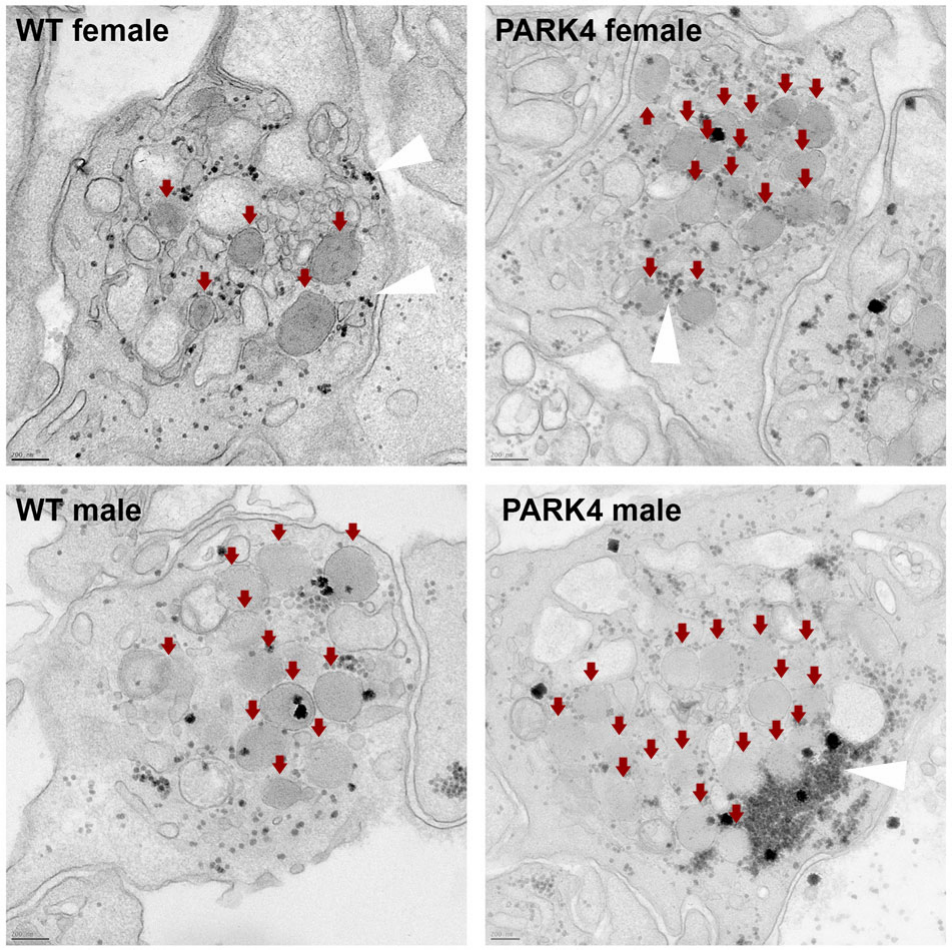
**Electron microscopy of blood platelets after stimulus-triggered degranulation is depicted, illustrating centrally clustered α-granules (red arrows) and glycogen granules (white arrowheads), but no detectable protein aggregates in PARK4 cases versus matched WT relatives.** Scale bars: 200 nm (*n*=2 control versus *n*=2 PARK4 individuals). WT, wild type.

Given that exogenous recombinant human *SNCA* and deletion of murine *Snca* are known to modulate the stimulus-triggered vesicular release from blood platelets (Park et al., 2002; Reheman et al., 2010), we investigated platelet function in two presymptomatic PARK4 heterozygotes (male aged 48 years and female aged 45 years) and two age- and sex-matched first degree control relatives (male aged 43 years and female aged 45 years) who could travel from Turkey to Germany. As revealed by flow cytometry, stimulation-induced degranulation increased the cell surface presence of the α-granule antigen CD62P (P-selectin) and the lysosomal CD63 antigen. In comparison to their control relatives and to the normal German population, these degranulation-dependent changes were diminished in presymptomatic PARK4 heterozygotes (Table 1). Electron microscopic analysis did not detect protein aggregates in the PARK4 platelets (Fig. 3). The presymptomatic PARK4 heterozygotes did not report any manifest blood coagulation disorder. These functional data might explain the expression dysregulation of the SNARE complex component *CPLX1* as a compensatory effort within the platelet activation pathway.

**Table 1.**
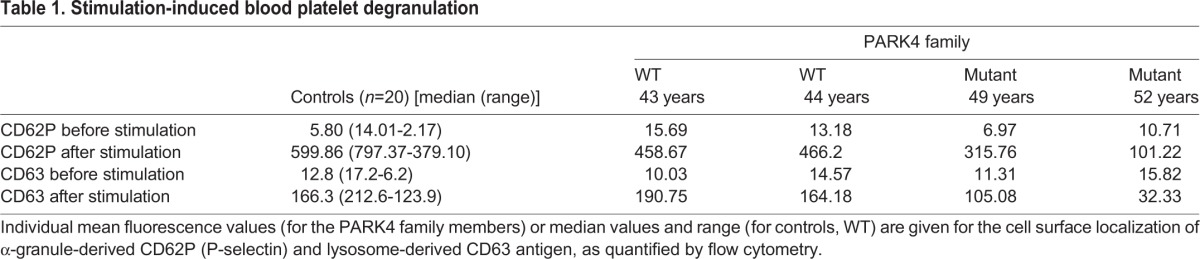
**Stimulation-induced blood platelet degranulation**

### Global RNA profiling of blood from presymptomatic PARK4 heterozygotes detects several strongly upregulated pathways

Global expression profiling by RNA-seq in blood samples from five presymptomatic PARK4 heterozygotes (male aged 53 years, female aged 59 years, male aged 50 years, female aged 47 years and male aged 50 years) versus five age- and sex-matched controls (male aged 45 years, female aged 54 years, male aged 57 years, female aged 42 years and male aged 42 years) was used to identify additional molecular effects of *SNCA* gene duplication. All data were deposited at the European Nucleotide Archive (ENA) public database (accession number PRJEB8960), and a table containing the ranked gene list of expression changes is provided in Table S1. In order to identify pathway dysregulations, the data were assessed bioinformatically with Gene Set Enrichment Analysis (GSEA). Downregulated pathways were not apparent, but several strong upregulations (with *P*-values=0.0 and *q*-values=0.0) were documented and concerned cytokine signaling in the immune system (normalized enrichment score, NES 4.8), adaptive immune system (NES 4.5), hemostasis (NES 4.4), lysosome (NES 4.3), platelet activation signaling and aggregation (NES 4.1), innate immune system (NES 4.0), endocytosis (NES 3.9), Toll-like receptor signaling (NES 3.3), metabolism of lipids and lipoproteins (NES 3.3) and extracellular region (NES 2.9). These unbiased findings are in excellent agreement with the known features and functions of SNCA in blood, such as altered coagulation and altered immune competence, and might reflect a compensatory increase in the well-established lysosomal degradation of SNCA (Westbroek et al., 2011).

### Blood qPCR shows an increase in *GZMH*, *SPP1* and *PLTP* in presymptomatic PARK4, but cannot distinguish prodromal multifactorial PD (RBD), whereas *CPLX1* is useful as a biomarker in PARK4 and RBD

#### Studies on presymptomatic PARK4 heterozygotes

**Fig. 4.**
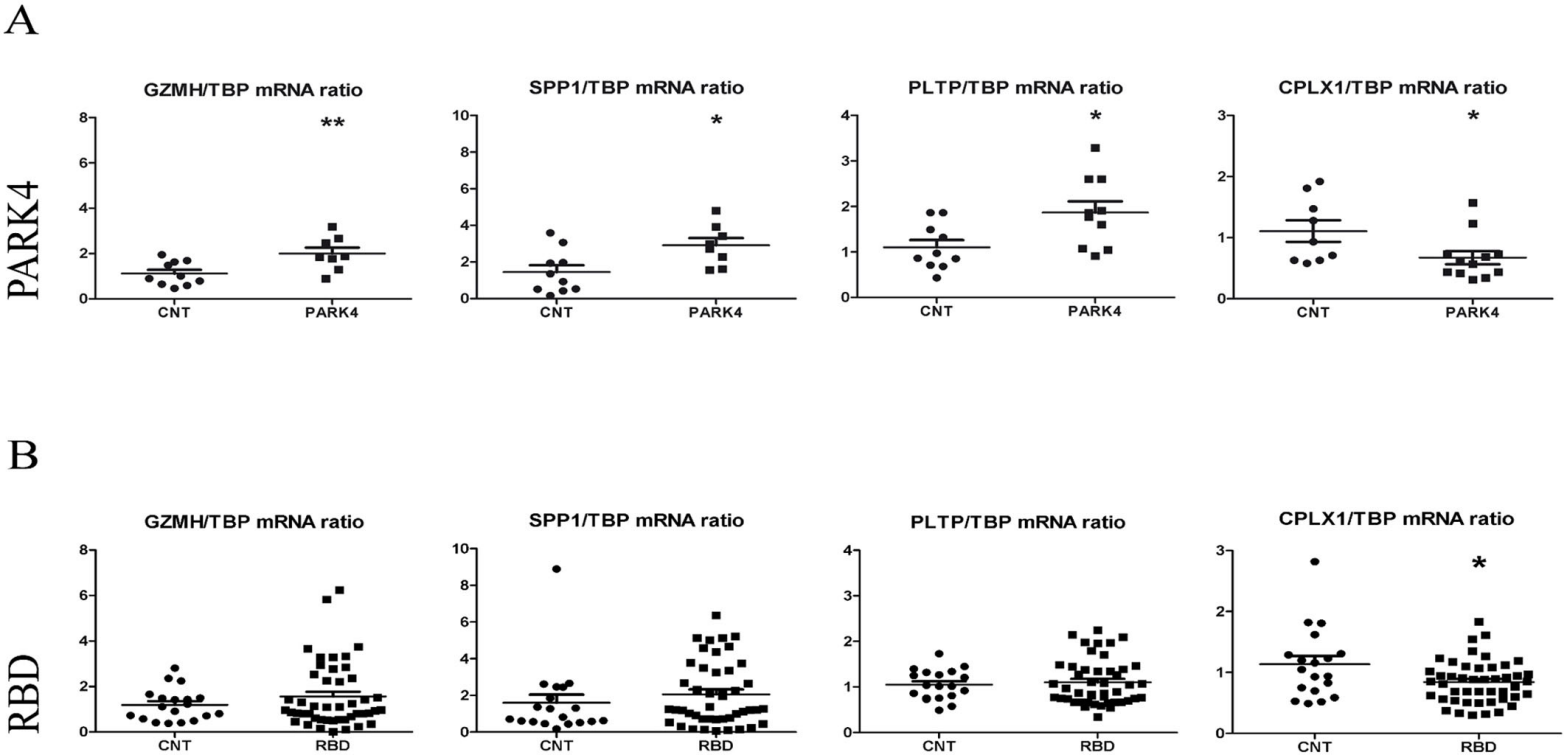
**Blood RNA-seq validation.** (A,B) mRNA levels assessed by qPCR in PARK4 (A) show downregulation of *CPLX1* and upregulation of *GZMH*, *SPP1* and *PLTP* (*n*=9 control versus 12 PARK4 individuals), and in RBD (B) show significant downregulation only for *CPLX1*, an upregulation for *SPP1* only after removal of one outlier value among controls (*n*=19 controls versus *n*=46 RBD cases) and no relevant changes for *GZMH* and *PLTP*. The individual value plots show the mean and s.e.m. *P<0.05, **P<0.01.

Particularly strong effects within the lysosome, immune and lipid pathways were studied as promising biomarker candidates for PARK4. We decided to validate the strong *GZMH* transcript upregulation (to 204%, *P*=0.04 in Student's paired *t*-test) by the independent qPCR technique in the same individuals plus additional members of the PARK4 pedigree, because the lysosomal enzymes cathepsin D and B, but not cathepsin G-like 2 (or granzyme H, GZMH) were previously implicated in degradation versus aggregation of SNCA (Crabtree et al., 2014; Tsujimura et al., 2014). The similar strong upregulation (to 244%, *P*=0.01) of the immunity regulator osteopontin (*SPP1*) transcript was assessed, because osteopontin levels were previously identified as a biomarker of PD in blood serum, cerebrospinal fluid, microglia and affected neurons (Iczkiewicz et al., 2006; Maetzler et al., 2007; Shi et al., 2015). The similar strong upregulation (to 189%, *P*=0.08) of the phospholipid transfer protein (*PLTP*) transcript was assessed, because PLTP modifies ataxia, Alzheimer's disease and tau phosphorylation and serves as lipopolysaccharide interactor (Dong et al., 2009; Gautier and Lagrost, 2011; Albers et al., 2012). The qPCR analysis confirmed significant increases for both transcripts (to 179% with *P*=0.009 for *GZMH*; to 201% with *P*=0.016 for *SPP1*; Fig. 4A). In comparison, the recently published longitudinally dynamic biomarkers of PD in blood, *HNF4A* and *PTBP1*, did not show significantly altered levels at this prodromal stage (*n*=9 control versus *n*=12 PARK4 individuals).

#### Studies on individuals with RBD but asymptomatic for parkinsonian motor symptoms

To investigate whether these candidate biomarkers might also be useful for risk prediction in individuals without a PD family history who manifest symptoms that might represent incipient PD (Postuma et al., 2012), we collected blood RNA from individuals with RBD after overnight fasting. The qPCR analysis of 46 individuals with RBD (mean age 65 years, range 34-83 years, with 36 males; since sampling, five individuals have converted to PD and one to Lewy body dementia, without DATscan confirmation) versus 19 matched controls (mean age 61 years, range 30-74 years, with 12 males) failed to detect a significant increase in *GZMH* (*P*=0.29), *SPP1* (*P*=0.35; but upon exclusion of one exceptionally high value among controls, an upregulation to 174% with *P*=0.04 was observed) and *PLTP* (*P*=0.66). By contrast, a significant downregulation was confirmed for the *CPLX1* transcript (to 74%, *P*=0.015; upon exclusion of one exceptionally high value among controls, a statistical trend towards downregulation with *P*=0.06 remained; Fig. 4B).

To define the stability of *CPLX1* mRNA levels in blood as a biomarker further, their dependence on sex and age was investigated. When the younger control individuals from Turkey were combined with the older control individuals from Central Europe, the expression did not show a significant correlation with age in linear regression analysis (*P*=0.33). No deviation from linearity was detectable in the runs test (*P*=0.53). There was no dependence on sex (*P*=0.50). The PARK4 cohort showed a trend towards reduced *CPLX1* values at older age (*P*=0.08) and a trend towards lower values in females (*P*=0.08).

Overall, the blood qPCR observations validate the RNA-seq findings and suggest that *GZMH*, *SPP1* and *PLTP* upregulations are indeed strong effects of PD pathogenesis. However, they might not be specific enough for the risk diagnostics of sporadic multifactorial PD, whereas the comparatively subtle *CPLX1* downregulation is useful in large populations with parallel sample processing, even if its fold change is too small for individual diagnostics.

### Reduced *CPLX1* mRNA in human *SNCA*-transfected neuroblastoma

**Fig. 5.**
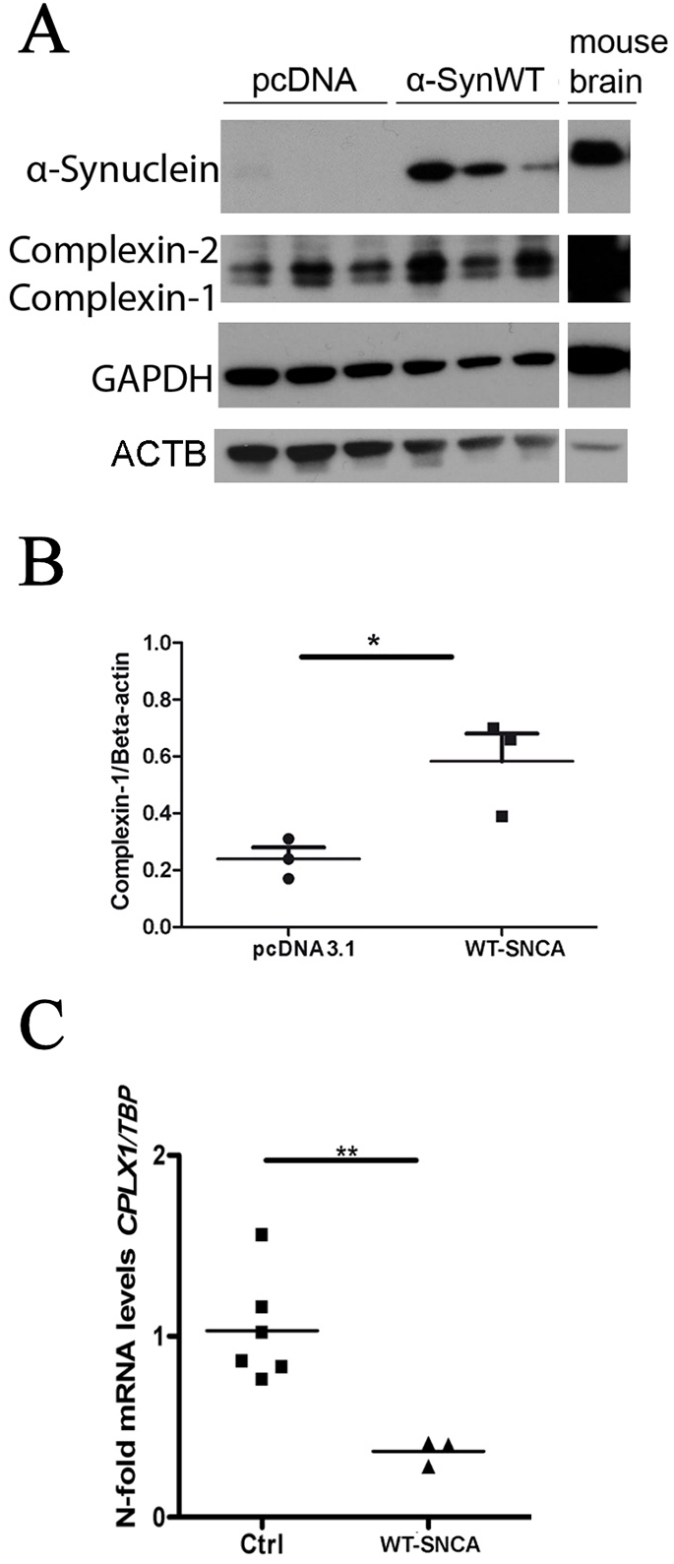
**Accumulation of complexin 1 protein and downregulation of *CPLX1* mRNA levels after overexpression of WT SNCA in SH-SY5Y cells.** Human SH-SY5Y neuroblastoma cells were transiently transfected with plasmid pcDNA3.1(+) as an empty vector control or WT SNCA. (A) Immunoblot confirmation of successful SNCA overexpression and of the effect on complexin 1 protein levels on the second day after transfection, using GAPDH and β-actin as the loading controls (*n*=3 versus *n*=3 versus *n*=3 in independent experiments). (B) Densitometric quantification of complexin 1 (antibody from Synaptic systems) versus ACTB ratios, normalized against control, in a scattergram analysis with *t*-test. (C) Analysis of corresponding *CPLX1* mRNA levels at 2 days after transfection. Asterisks indicate statistical significance versus control group (*n*=6 versus *n*=3 versus *n*=12). *P<0.05, **P<0.01. ACTB, β-actin; α-Syn, α-synuclein; WT, wild type.

To further evaluate the SNCA-triggered expression changes, we transiently overexpressed wild-type SNCA in human SH-SY5Y neuroblastoma cells, a widely used PD cell model with catecholaminergic properties. A downregulation of *CPLX1* mRNA in parallel to accumulation of complexin 1 protein occurred within 2 days after transfection (Fig. 5). This demonstrates that these effects are initiated early in neural cells. The observed upregulation of complexin 1 protein in radioimmunoprecipitation assay (RIPA) buffer fractions of these cells after 2 days contrasts with the downregulation of complexin 1 protein in 8 M urea fractions that was recently observed in the prefrontal cortex of autopsied individuals who had PD in old age (Dumitriu et al., 2016) and might be explained by altered CPLX1 solubility, by progression from acute to chronic SNCA gain of function or by other differences between neuroblastoma cells versus cortical postmitotic neurons.

### Does complexin 1 protein aggregate in the midbrain in PD?

**Fig. 6.**
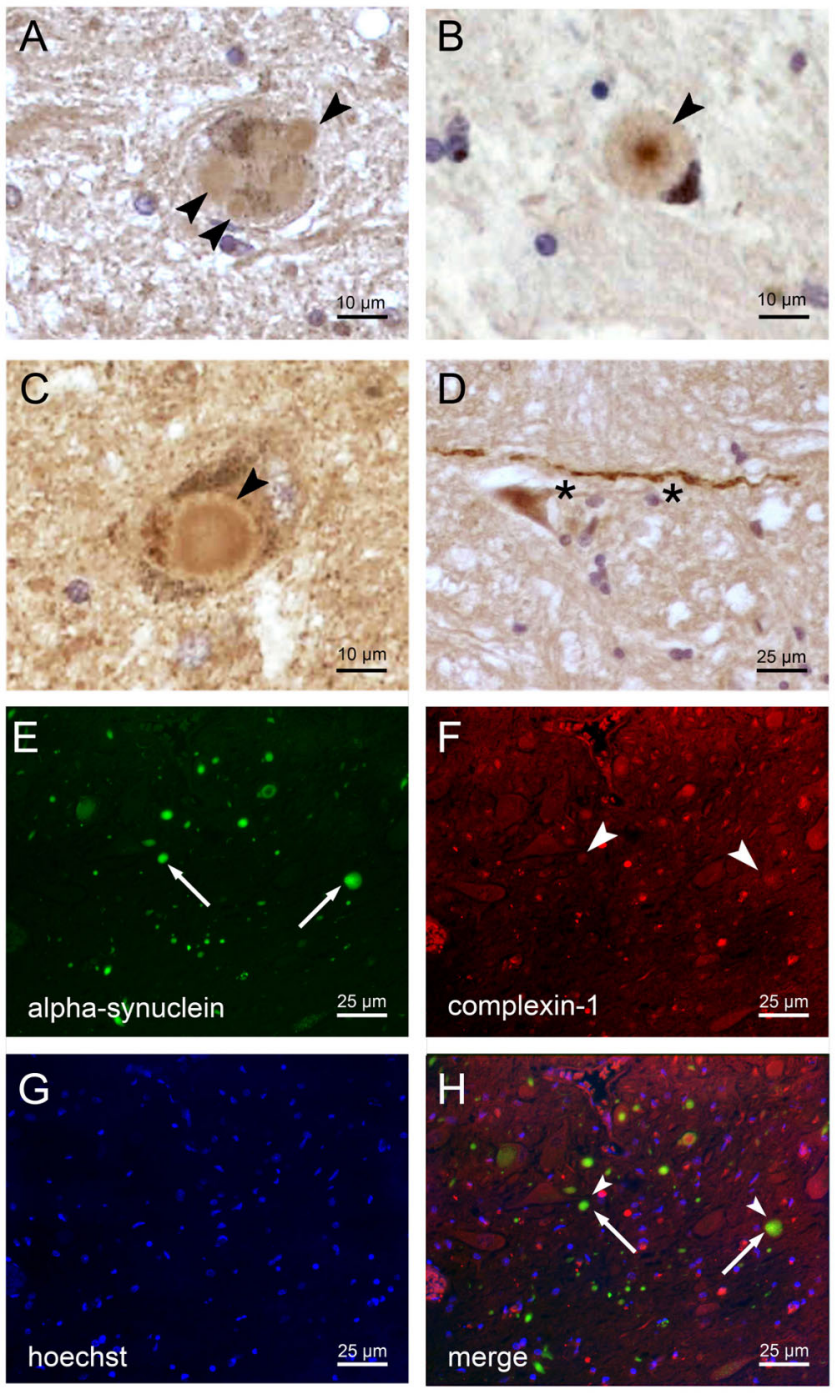
**PD midbrain autopsies contain cytoplasmic and neuritic aggregates.** Tissue sections (5 μm thick) from individuals with PD were stained with anti-complexin 1 (Acris) or double stained with anti-complexin 1 and anti- SNCA antibodies. (A-C) Neurons in the substantia nigra exhibiting Lewy body-like structures mildly stained with anti-complexin 1 (arrowheads). (D) Complexin 1 immunopositive Lewy neurite-like structure (asterisks) in the medulla at the level of the motor vagus nucleus. (E-H) Double immunostaining depicting possible co-localization of α-synuclein (SNCA; green) and complexin 1 (red), only after maximal exposure and contrast adjustment in the red channel. Sections were counterstained with Hoechst dye.

Protein levels of SNCA and complexin 1 are increased in midbrain tissue from individuals with idiopathic PD and from mouse models of synucleinopathy (Basso et al., 2004; Gispert et al., 2015b). Such accumulations might be attributable to elevated gene dosage with increased transcript levels or to impaired degradation during an aggregation process. The latter mechanism might be responsible for the decreased *SNCA* transcript levels that were described in some individuals with PD (Dächsel et al., 2007). Several other components of the SNARE complex that mediate membrane fusion and vesicle exocytosis, namely SNAP25, syntaxin 1 and synaptobrevin 2 (VAMP2), are contained in Lewy bodies and are depleted from surrounding neural tissue in PD (Garcia-Reitbock et al., 2010; Mukaetova-Ladinska et al., 2013). To test whether complexin 1 is aggregating within Lewy bodies, as was previously observed for SNCA and 14-3-3, we performed immunohistochemical analyses of midbrains from individuals with PD. A low-titer antibody (Acris) detecting complexin 1 with high specificity stained aggregates in neurites and neuronal perikarya of fixed tissue after antigen retrieval (Fig. 6A-D). Double immunofluorescence revealed co-localization of α-synuclein and complexin 1 in fixed tissue only after maximal exposure and contrast adjustment of the channel depicting complexin 1 (Fig. 6E-H). These observations might support the notion that complexin 1 is sequestered into Lewy bodies, but of course the evidence must be considered with caution given that numerous proteins might be entrapped in aggregates without a specific role in pathogenesis.

The recent report of a 20% reduction of CPLX1 protein in 8 M urea fractions of prefrontal cortex from autopsied individuals with PD (Dumitriu et al., 2016) is also compatible with a sequestration of complexin 1 into Lewy bodies. Given that we documented a 33% reduction of *CPLX1* expression in blood of presymptomatic PARK4 individuals, the dysregulation appears to be relatively stable over decades and might not be useful as a biomarker of disease progression from the prodromal to the final stages of PD. Therefore, extensive investigations of *CPLX1* expression in individuals with PD of varying severity were not attempted.

### *CPLX1* gene variant enhances PD risk

It remains unclear whether *CPLX1* modulation is neuroprotective or instead contributes to pathogenesis. Interestingly, the *CPLX1* gene is encoded within a locus of confirmed association with PD risk according to GWAS meta-analyses (Lill et al., 2012). The corresponding PD risk haplotype, with an odds ratio of 1.45 at chromosome 4p16.3, spanned the genes *PCGF3/LOC100129917/CPLX1/GAK/TMEM175/DGKQ*. The GWAS approach cannot reliably dissect such loci further, although it can detect relative disease risks as low as 1.1-fold. In the latest meta-analysis of GWAS studies in PD (Nalls et al., 2014), two single nucleotide polymorphisms (SNPs) in the intron 1 of *CPLX1* directly next to the *GAK* gene reached genome-wide significance (rs76444973 and rs34006598), but it is difficult to predict how these variants would influence the function of CPLX1 protein. We therefore used a complementary approach and searched for a low-frequency *CPLX1* gene variant, which shows its risk association already in small case-control collectives and explains a substantial part of the disease association contained in the locus. For this purpose, 360 random individuals with idiopathic general (non-monogenic) PD and 358 controls were studied regarding several candidate SNPs outside the linkage disequilibrium block around *GAK*, within the *CPLX1* gene 3′-UTR. The selected SNPs show minor allele frequencies between 0.1 and 0.5 in Caucasian populations and were available as TaqMan genotyping assays. Significant association with PD risk was observed for the G allele of the SNP rs10794536 (Table 2), with an odds ratio of 1.33. This 3′-UTR variant might influence the stability of *CPLX1* mRNA. These data suggest that complexin 1 not only serves as a downstream marker of the physiological α-synuclein function, but also modulates PD risk.

**Table 2.**
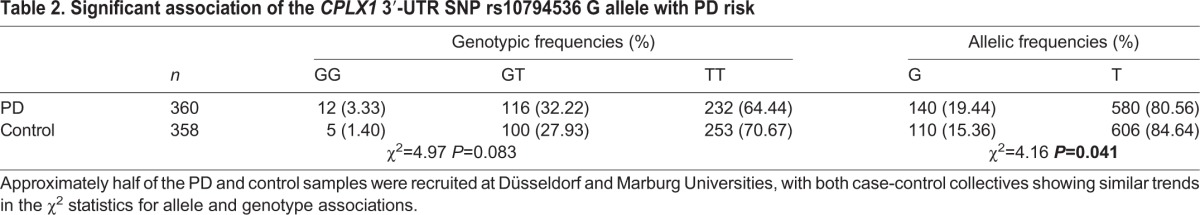
**Significant association of the *CPLX1* 3′-UTR SNP rs10794536 G allele with PD risk**

### *Cplx1* gene ablation upregulates α-synuclein levels

**Fig. 7.**
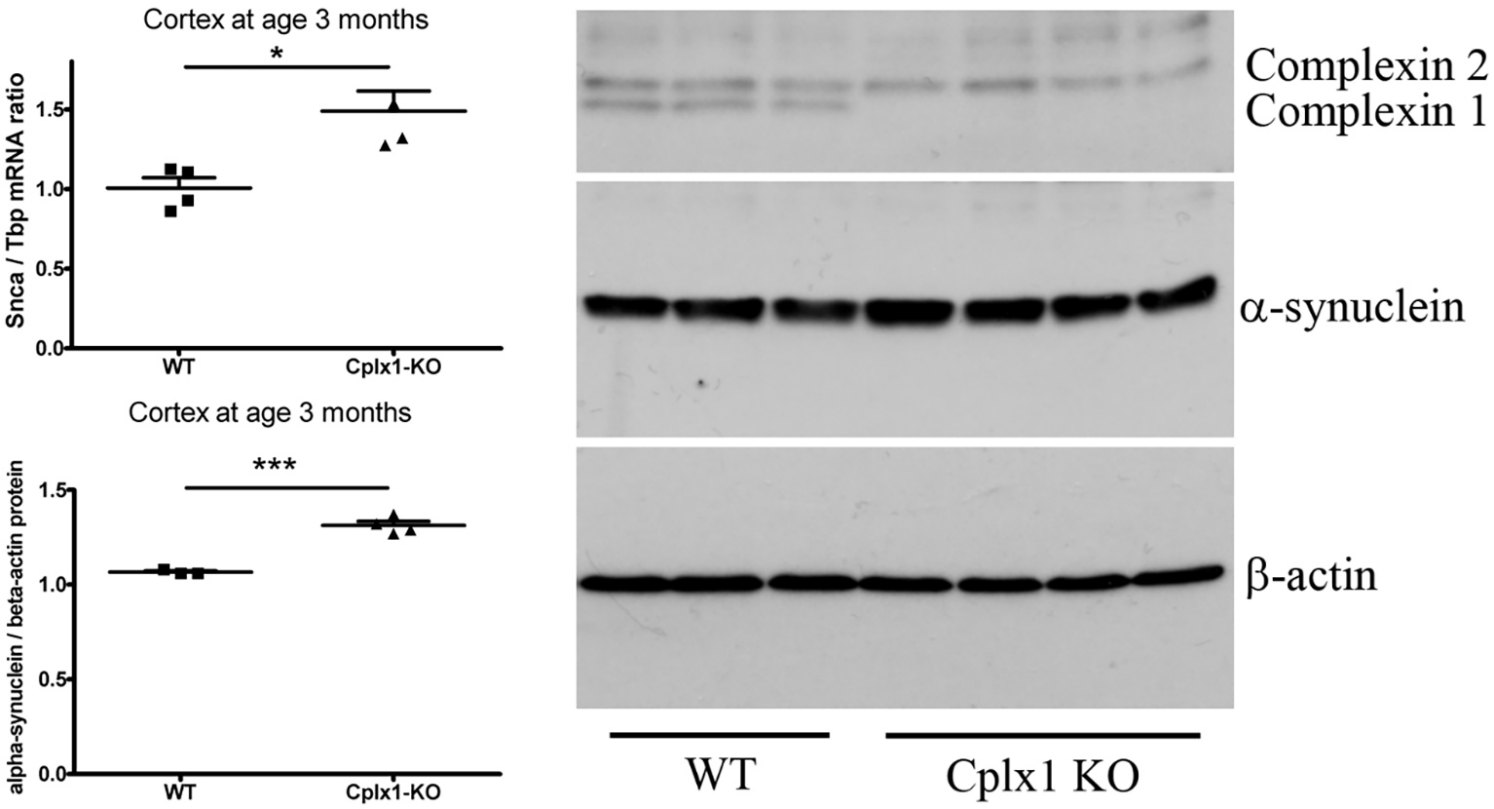
**Elevated SNCA mRNA and protein levels in adult mouse *Cplx1*^−/−^ cortex.** Normalized fold changes are shown for qPCR (*n*=4 versus *n*=4, left above) and immunoblot quantitation (*n*=3 versus *n*=4, left below) in scattergrams, together with representative scans of western blots for complexin 1/2, α-synuclein and β-actin (right). *P<0.05; ***P<0.001.

In order to determine whether complexin 1 loss of function enhances or alleviates the susceptibility to PD, the relevant brain region was studied in a *Cplx1* null mouse. This mouse line was previously shown to have an early and strong cerebellar phenotype (Reim et al., 2001; Glynn et al., 2005). In addition, these mice display dystonia, shuffled walking and reduced novelty seeking, several signs that are characteristic effects of nigrostriatal dysfunction; intriguingly, these mice also display resting tremor, a diagnostic hallmark in humans for the onset of intermediate stage PD (Glynn et al., 2005). However, only cerebellar and not cerebral tissues have been investigated to date. Brains of the *Cplx1* null mice revealed significantly increased levels (1.3- to 1.5-fold) of *Snca* mRNA and SNCA protein by 3 months of age (Fig. 7). The data provide further evidence that complexin 1 levels are not only a downstream marker of SNCA function, but are also involved in SNCA abundance, apparently by reciprocal feedback regulation of expression.

## DISCUSSION

In summary, we report a new, exceptionally large PARK4 pedigree, provide proof of principle that the risk for a future manifestation of PD is reflected in the global transcriptome of blood, identify diverse pathways and, in particular, the levels of *SPP1*, *GZMH*, *PLTP* and *CPLX1* as biomarkers of pathogenesis, show subtle *CPLX1* downregulations to distinguish prodromal PD cohorts (presymptomatic *PARK4* heterozygotes and RBD individuals) from controls, and suggest that complexin 1 loss of function acts as a PD risk factor.

Twenty-five years ago, the characterization of the large Italian Contursi PARK1 kindred triggered research into PD genetics (Golbe et al., 1990; Polymeropoulos et al., 1996, 1997). Now, the cooperativity of the Turkish PARK4 pedigree is a unique opportunity for further studies into human synucleinopathy, such as brain imaging, and the existence of 12 presymptomatic heterozygotes of the *SNCA* gene duplication in this family might be crucial for the identification of a molecular signature of disease risk. Our deposition of PARK4 blood RNA-seq data at the ENA database aims to drive this effort, ensuring that PD risk, which is triggered by the genetically defined synucleinopathy of PARK4 in the Turkish pedigree and mirrored by specific pathways and molecular biomarkers, can now be compared with diverse ongoing data collections in individuals with manifest PD, usually without a family history and with multifactorial pathogenesis (Scherzer et al., 2007; Marek et al., 2011; Santiago and Potashkin, 2015).

It is unlikely that diagnostics of PD risk can be based on a single technique or a single molecular biomarker, so we evaluated the global transcriptome and the pathway dysregulations by GSEA bioinformatics. These approaches recently permitted us to identify three gene expression changes in dependence of SNCA function in brain tissue, concerning the chemotaxis factor *Lect1* (*Cnmd*) in one digenic and two monogenic PD mouse models (Gispert et al., 2015a) and the midbrain-selective transcription factor *Foxp1* and the SNARE complex component *Cplx1* in a synucleinopathy model and SNCA-deficient mouse mutants (Gispert et al., 2015b). Although we were unable to detect substantial *FOXP1* and *LECT1* expression in human fresh whole blood PAXgene samples, despite strong signals having been reported in microarray studies of purified peripheral mononuclear blood cells, we found mRNA complexin 1 levels to be downregulated in whole blood in the Turkish PARK4 pedigree. The observation that increased abundance of SNCA, a known modulator of the SNARE complex and of vesicle dynamics, modifies the expression of another SNARE interactor, CPLX1, led us to hypothesize that altered physiological functions of SNCA at the earliest stages of the accumulation and aggregation process will trigger immediate molecular and cellular responses in a subtle but cumulative manner, which might be exploited as biomarkers. This notion was supported by GSEA pathway dysregulations for the metabolism of lipids and lipoproteins and for endocytosis in response to the PARK4 mutation in the lipid-binding endocytosis modulator SNCA (Diao et al., 2013). Furthermore, this concept was also substantiated by dysregulated hemostasis and platelet activation pathways, with corresponding preliminary documentation of impaired platelet degranulation in PARK4 blood, in the absence of detectable protein aggregates, in excellent agreement with previous publications *in vitro* and in mice on a role of SNCA in platelet activation (Park et al., 2002; Reheman et al., 2010).

Our efforts to identify additional molecular biomarkers with stronger effect sizes focused on *GZMH* levels as representative of the lysosome pathway upregulation, on *SPP1* levels as representative of the immunity pathway upregulation, and on *PLTP* levels as representative of the lipid metabolism pathway. The data clearly confirmed their role as biomarkers of PARK4, at least in this Turkish pedigree, so it might now be interesting to test whether progressive stages of synucleinopathy can be correlated with expression levels of these factors or of other pathway components, whether these factors represent molecular targets of neuroprotective therapies or are risk factors themselves. Interestingly, a moderate mRNA upregulation for the lysosomal enzyme glucocerebrosidase (GBA; log_2_ fold change=0.31) was notable in the PARK4 blood RNA-seq GSEA; GBA is known to contribute to SNCA degradation, and its mutations act as modifiers of PD risk (Yap et al., 2011; Sidransky and Lopez, 2012; Fishbein et al., 2014). But there are many triggers of lysosomal induction and immunity responses beyond SNCA accumulation and PD, and this probably explains why *GZMH* and *SPP1* upregulations are not specific for indivduals with RBD but are also detectable among some control individuals.

Despite the small effect size of *CPLX1* mRNA downregulation, this observation reproducibly distinguished cohorts of presymptomatic PARK4 heterozygotes in blood, of individuals with RBD in blood, and neuroblastoma cells after SNCA transfection. The corresponding protein levels are difficult to quantify in blood because of the abundance of hemoglobin, albumin and fibrinogen, and because small fold changes are difficult to detect with antibody-dependent techniques and non-linear enzyme kinetics. CPLX1 protein accumulated in neuroblastoma cells (proteins extracted with RIPA buffer) in our experiments and is known to accumulate together with SNCA in midbrain tissue (proteins extracted with 7 M urea, 2 M thiourea and 4% CHAPS), whereas it is decreased in the prefrontal cortex of individuals with sporadic PD (proteins extracted with 8 M urea; Basso et al., 2004; Dumitriu et al., 2016). Thus, the abundance of CPLX1 protein in fractions of different solubility appears to vary between different brain areas or in dependence on the progression of SNCA aggregation. We speculate that the accumulation of insoluble SNCA through protein interaction sequesters CPLX1 protein, leading to its accumulation in solubility, later in insolubility, whereas a homeostatic negative autoregulation reduces the *CPLX1* mRNA levels. The decrease of *CPLX1* mRNA has clear advantages as risk biomarker in peripheral tissue, whereas complexin 1 protein redistribution might become a biomarker of disease progression in nervous tissue. The formation of Lewy bodies has been shown to deplete SNARE complex components from neurons, although it is technically difficult to demonstrate their presence within the protein inclusion bodies (Garcia-Reitbock et al., 2010; Mukaetova-Ladinska et al., 2013). The unequivocal co-localization of SNCA and CPLX1 in aggregates is challenging. Co-immunoprecipitation studies failed to detect a direct association, but the high detergent conditions needed to enrich SNCA oligomers and fibrils would not leave protein associations intact.

Thus, instead of pursuing the physical interaction between SNCA and CPLX1 proteins, we decided to assess their genetic interaction further. Two lines of evidence, (1) the identification of the *CPLX1* gene variant rs10794536 that is associated with PD risk in a relatively small case-control study, and (2) the demonstration that genetic ablation of *Cplx1* in mouse brain leads to an elevation of *SNCA* levels that is comparable to PARK4, both argue that complexin 1 also has a role as risk factor in PD, not only a role as biomarker. The role of complexin 1 appears to extend beyond monogenic synucleinopathy cases into sporadic PD, in view of (1) its accumulation in mass-spectrometry proteomics of PD midbrain autopsies, (2) the GWAS meta-analyses of risk loci for sporadic PD, and (3) our findings of *CPLX1* blood level downregulation in RBD cases.

It is noteworthy in this context that a recent report showed a knock-in mutation in another SNARE protein, unphosphorylatable S187A-substituted SNAP25, to trigger increased abundance and mislocalization of SNCA, together with SNARE assembly problems and presynaptic dilatation, but without neurodegeneration until the mice were 1 year old. These authors concluded that dysfunctions at the SNARE complex in presynapses might induce elevated SNCA abundance and thus lead to an insidious neurodegenerative process (Nakata et al., 2012; Yasuda et al., 2013). This scenario is in good agreement with the recently identified genetic risk factors for idiopathic PD in individuals without monogenic traits. The reproducible GWAS loci of PD risk (Lill et al., 2012; Nalls et al., 2014) are surprisingly enriched in genes relevant for vesicle dynamics, such as the *SYT11* (encoding synaptotagmin 11) and *RAB25* genes at the chromosome 1q21.1-22 PD risk locus, the *RAB29* gene at the chromosome 1q32 PARK16 locus, the *NSF* gene (encoding N-ethylmaleimide sensitive factor) within the risk haplotype at the chromosome 17q21 PD risk locus named after *MAPT*, and the *SYT4* gene (encoding synaptotagmin 4) at the chromosome 18q12.3 PD risk locus named after *RIT2*. A remaining major GWAS PD risk locus contains the *LRRK2* gene, which is responsible for autosomal dominant PARK8 and encodes a protein that modulates synaptic vesicle cycling and that interacts with syntaxin 1B/NSF/clathrin/AP2 (Piccoli et al., 2011). Thus, the *SNCA* and *CPLX1* 3′-UTR variants might be representative for various SNARE/RAB vesicle cycle alterations that modulate PD risk.

Overall, our initial characterization of an exceptionally large PARK4 pedigree from Turkey provided insights into the prodromal stage of monogenic synucleinopathy, defining a molecular signature in the blood transcriptome that might be relevant for predictive diagnostics in groups of at-risk individuals. Although several differences in expression between groups with presymptomatic PD versus controls were identified with statistical significance, individual differences at present would be insufficient for predictive diagnostics. Most importantly, the data identify *CPLX1* as a biomarker and modifier gene of PD risk.

## MATERIALS AND METHODS

### Neurological examination

Neurological examination of members of the PARK4 family, which originated in the North Eastern region of Turkey, to document clinical manifestation or presymptomatic PARK4 heterozygote status adhered to the UK PD Society Brain Bank criteria (Hughes et al., 1992).

### Genotyping

Genomic DNA from blood samples in EDTA tubes stored at +4°C was extracted with MagNA Pure Systems (Roche), after informed written consent and genetic counseling, with approval from the Ethics Commission of Boğaziçi University Istanbul. The molecular genetic diagnosis of PARK4 in an index individual with PD from this family (female aged 51 years with UPDRS (unified Parkinson's disease rating scale) score 7 after a disease duration of 7 years, showing rigidity, resting tremor and bradykinesia, having 60 months of levodopa treatment with some benefit, but dyskinesias) was originally made by semi-quantitative multiplex PCR and multiplex ligation-dependent probe amplification (P051 kit) of *SNCA* exons 3 and 4 in comparison to *Parkin* and *TTR* exons 4, then extended to examine 22 pedigree members. Demonstration of *SNCA* gene duplication was complemented by the detection of the associated 4q21-3-4q22.3 haplotype of 17 microsatellite markers (D4S2691, D4S2409, D4S2462, D4S2622, D4S1542, D4S2929, D4S2371, D4S2461, D4S2304, D4S3459, D4S3457, D4S1544, D4S410, D4S1089, D4S414, D4S2404 and D4S2364) across 11 Mb around the *SNCA* locus in 12 pedigree members to demonstrate co-segregation. Further genotyping experiments were performed for the PARK4 family members using DNA probes specific to the *SNCA* gene and the quantitative real-time PCR method. Expression levels of the β-actin, *GAPDH* and *TBP* genes were separately used as endogenous controls for normalization. The gene expression fold changes were analyzed according to the 2^−ΔΔCt^ method (Livak and Schmittgen, 2001).

### Molecular karyotyping

Two PARK4 blood samples were assessed for copy number variation using a SurePrint G3 Human CGH (comparative genomic hybridization) microarray 2×400 K design 021850 (Agilent Technologies). Agilent's labeling kit was used, and all procedures were carried out according to the manufacturer's instructions. Scanning was performed on an Agilent microarray scanner and raw data were processed by Feature Extraction 9.5. Deleted and amplified regions were determined on Agilent's Genomic Workbench Standard Edition 5.0.14. A minimum of four consecutive probes had to be affected to make a decision. The aberration detection threshold of the ADM-2 algorithm was set to 5.9.

### Whole blood transcriptomics and protein analysis

After overnight fasting of the individuals, whole blood samples from the PARK4 family were taken (after informed written consent and with approval from the Ethics Commission of Boğaziçi University Istanbul) into PAXgene (Qiagen) and EDTA tubes. Also after overnight fasting, whole blood samples were collected into PAXgene tubes from a RBD cohort comprising 46 affected individuals and 19 healthy relatives (individuals with narcolepsy were excluded), after written informed consent and with approval from the Ethics Commission at Marburg University. Samples from affected individuals and controls were processed in parallel, and after 2 h incubation of the PAXgene tubes at room temperature, they were stored at −80°C until the RNA was extracted after a further overnight room temperature incubation, using the corresponding PAXgene kit. The isolated RNA was cleaned up by DNAse-I amplification grade (Invitrogen) and converted to cDNA by SuperScript III Reverse Transcriptase (Invitrogen). The quality and quantity of the cDNA were determined by spectrophotometry and RNA integrity numbers (Agilent Bioanalyzer). Quantitative RT-PCR was performed with TaqMan assays (Applied Biosystems) Hs01018439_s1 (*GPRIN3*), Hs00201182_m1 (*MMRN1*), Hs00411197_m1 (*LRRK2*), Hs01084510_m1 (*GIGYF2*), Hs00223032_m1 (*ATP13A*), Hs01026447_m1 (*ATXN3*), Hs00697109_m1 (*DJ-1*), Hs00260868_m1 (*PINK1*), Hs00832556_sH (*ST13*), Hs01038318_m1 (*PARK2*), Hs00201825_m1 (*FBX07*), Hs00188233_m1 (*UCHL1*), Hs00164683_m1 (*GBA*), Hs00234883_m1 (*HTRA2*), Hs00185926_m1 (*PLA2G6*), Hs00608185_m1 (*SNCB*), Hs00705917_s1 (*YWHAG*), Hs01103386_m1 (*SNCA*), HS00793604_m1 (*YWHAB*), HS00356749_g1 (*YWHAE*), Hs00362510_m1 (*CPLX1*), Hs00277212_m1 (*GZMH*), Hs00959010_m1 (*SPP1*), Hs00272126_m1 (*PLTP*), Hs00230853_m1 (*HNF4A*) and Hs00914687_g1 (*PTBP1*). After studies of various loading controls (e.g. *GAPDH*, *ACTN*, *RPL13A*, *TBP*, *SDH* and *YWHA2*) versus *SNCA* expression in whole blood, Hs99999910_m1 (*TBP*) was observed to show the smallest variance and was therefore included as the housekeeping control for each expression test. A total of 25 ng cDNA was used in each reaction. The PCR conditions were 2 min at 50°C, 10 min at 95°C, 40 cycles of 15 s at 95°C and 1 min at 60°C. The gene expression changes were analyzed according to the 2^−ΔΔCt^ method (Livak and Schmittgen, 2001).

### Whole blood protein analysis

For protein extraction from the EDTA tubes, 300 μl blood were lysed with an equal amount of 1% SDS-RIPA buffer [50 mM Tris-HCl (pH 8.0), 150 mM NaCl, 1 mM EDTA, 1 mM EGTA, 1% Igepal CA-630 (Sigma), 0.5% sodium deoxycholate, 0.1% SDS, 1 mM PMSF and one tablet Complete Protease Inhibitor Cocktail (Roche)] and sonicated for 10 s. The blood lysates were rotated at 4°C for 30 min and centrifuged at 4°C for 30 min. The supernatants were depleted in hemoglobin content using a commercial kit (HemogloBind, Biotech) following the manufacturer's instructions. Protein concentration was determined using the BCA protein assay kit (Thermo Fisher Scientific). For immunoblotting, proteins were diluted to 4 μg/μl and mixed with an equal amount of loading buffer (250 mM Tris-HCl pH 6.9, 20% glycerol, 4% SDS, 10% mercaptoethanol and 0.005% Bromophenol Blue). Samples were incubated at 65°C for 16 h before being loaded on the 12% polyacrylamide gels and transferred to PVDF (polyvinylidene fluoride) membranes. The membrane was blocked in 5% milk powder and incubated with primary antibodies against α-synuclein (1:1000, BD 610786), β-synuclein (1:1000, Abcam ab6165 and Upstate 36-009), complexin 1 (1:500, Acris AP51050PU-N) and β-actin (1:10,000, Sigma, A5441), visualizing the signals by the ECL method. Densitometric analysis was carried out using ImageJ software.

### Blood platelet flow cytometry

After applying a light tourniquet, which was immediately released after venipuncture, blood was drawn using a 21-gauge butterfly needle. Blood was collected in 4.5 ml coagulation tubes containing 0.106 mol/l sodium citrate (Sarstedt AG) for flow cytometry evaluations. The following directly conjugated monoclonal mouse anti-human antibodies were obtained from BD Biosciences: phycoerythrin (PE)-conjugated anti-CD62P, PE-conjugated anti-CD63 and (Perc-CP)-conjugated anti-CD61. Platelet immunostaining was performed as follows: 20 μl diluted citrated whole blood [1:10, Dulbecco's modified phosphate saline without Ca^2+^ and Mg^2+^ (DBPS), Gibco BRL] was incubated with saturating concentrations of monoclonal antibodies for 15 min at room temperature. Thereafter, samples were diluted with 1 ml DPBS and analyzed immediately by FACS Calibur flow cytometer (BD Biosciences). Platelets were identified through CD61 positivity and their characteristic light scattering. Each analysis was performed on 5000 platelets. Quantification of surface expression of given antigens was obtained using CellquestPro software (BD Biosciences). The mean fluorescence intensity was used to define molecule expressions on the platelet surface. Results were compared with 20 healthy Central European volunteers (control group). To characterize the α-granule and lysosome release response upon platelet activation, 20 μl of 1:10 diluted citrated whole blood was stimulated with thrombin receptor agonist peptide (TRAP-6, final concentration 10 µmol/l, Roche) and DPBS (control) for 10 min at room temperature. Platelets were immunostained as described above.

### Platelet electron microscopy

Approximately 16 ml of blood was collected in 8.2 ml coagulation tubes containing 0.106 mol/l sodium citrate (Sarstedt AG). Fresh platelet-rich plasma (PRP) was prepared by centrifugation at 140 ***g*** for 10 min at room temperature. After being stored for 30 min, PRP samples were stimulated with TRAP-6, final concentration 30 µmol/l, for 30 s. Fixation of suspended platelets was accomplished by combining the sample with an equal volume of 0.1% glutaraldehyde in White's saline [a 10% solution of a 1:1 mixture of the following: (1) 2.4 mmol/l NaCl, 0.1 mmol/l KCH, 46 mmol/l MgSO_4_ and 64 mmol/l Ca(NO_3_)_2_.4H_2_O; and (2) 0.136 mol/l NaHCO_3_, 8.4 mmol/l NaH_2_PO and 0.1 g/l of phenol red, pH 7.4]. After 15 min the samples were centrifuged to pellets, washed and resuspended in 3% glutaraldehyde in the same buffer, resuspended and maintained at 4°C for 30 min, then sedimented to pellets. The supernatant was removed and replaced with 1% osmic acid in distilled water containing 1.5% potassium ferrocyanide for 1 h at 4°C. All samples were dehydrated in a graded series of alcohols and embedded in Epon. Thin sections of 100 nm were cut from the plastic blocks on a Leica ultramicrotome and examined after staining with uranyl acetate and lead citrate to enhance contrast. Samples were examined on a Technai F30 electron microscope at 300 kV equipped with a US 4000 camera.

### RNA-seq

Global blood RNA sequencing for the five PARK4 versus five age- and sex-matched healthy individuals was outsourced to Alacris Theranostics GmbH, Berlin, Germany. Strand-specific RNA sequencing libraries were prepared from 500 ng of total RNA according to the Illumina TruSeq stranded mRNA protocol. mRNA was selected using oligo(dT) Dynabeads. Sequencing was performed on the Illumina HiSeq2500 platform, using paired-end 2×50 bp sequencing mode. For each library, 40-50 million passed filter reads were obtained. Raw data were processed according to the manufacturer's instructions. Illumina's bcl2fastq software v1.8.2 was used for base calling and demultiplexing. The sequencing reads were mapped to the human genome reference GRCh37/hg19 using bwa (version 0.5.9, http://bio-bwa.sourceforge.net/). Exonic reads in correct genomic orientation, defined by Ensembl Release 73 (http://www.ensembl.org/index.html), were counted using custom Python scripts. Expression values were calculated using RPKM (reads per kilobase per million mapped reads) normalization with custom R scripts (Mortazavi et al., 2008). Given that we observed huge amounts of hemoglobin in the samples, reads on hemoglobin genes were removed before the expression analysis. Differential expression between disease and control samples was calculated with the R bioconductor package edgeR (Robinson et al., 2010; http://www.bioconductor.org/packages/release/bioc/html/edgeR.html), applying an exact test for overdispersed data. As an additional criterion to judge variance and restrict the number of biomarker candidates, a custom R script was used to calculate Student's paired *t*-tests between disease and control expression values for each gene.

### Bioinformatic systems biology

RNA-seq data from five presymptomatic PARK4 heterozygotes and five matched controls were analyzed to compute average fold changes for each gene and rank them according the *t*-test statistics. For duplicate entries, the maximal value was used. The ranked list of gene symbols thus generated was subjected to nonspecific filtering and assessed by GSEA using the Java-based version GSEA-P (Subramanian et al., 2005, 2007). Permutations were performed on gene sets owing to the low number of biological replicates. We used the c2 (online pathway databases, Pubmed publications, expert of domain knowledge), c5 (Gene Ontology categories) and cc (Gene Ontology cellular component) gene sets from the MSigDB database (v.4.0, May 2013, http://www.broadinstitute.org/gsea/msigdb/index.jsp) to analyze the data sets.

### Transient transfection of wild-type SNCA in SH-SY5Y cells

The open reading frames corresponding to wild-type SNCA were cloned into the plasmid pcDNA3.1(+) (Invitrogen), between the restriction sites *Kpn*I and *Not*I. Transient overexpression was performed by nucleofection using 1.5 million SH-SY5Y cells (recently authenticated and tested for contamination) and 2 µg of plasmid together with Amaxa nucleofector Kit V (Lonza). Forty-eight hours post-transfection, total RNA was isolated using the RNeasy kit (Qiagen), cDNA was synthesized using 1 µg of total RNA and relative mRNA levels were determined by qPCR using the TaqMan probes *SNCA* (Hs00240906_m1), *CPLX1* (Hs00362510_m1) and *TBP* (Hs99999910_m1) (Applied Biosystems). Relative mRNA levels were calculated using the 2^−ΔΔCt^ formula. Total protein was isolated with lysis buffer [Tris-HCl 137 mM pH 6.8, sodium dodecyl sulfate 4%, glycerol 20% and protease inhibitors 1X (Roche)]. Thirty micrograms of protein was resolved in a 4-12% gradient gel (Novex NP0322BOX), transferred to a PVDF membrane, and blocked with 5% skimmed milk in PBS-Tween 0.1%. Antibodies against the following proteins were used: α-synuclein (1:1000; Covance, SIG-39730-200); complexin 1+2 (1:1000; Synaptic systems, 122002); β-actin (1:1000; Sigma-Aldrich, A5441); GAPDH (1:1000; Calbiochem, CB1001).

### Brain autopsy processing and immunohistochemistry

Brain autopsy processing and immunohistochemistry were performed as described previously (Seidel et al., 2010a,b), using primary antibodies for complexin 1 (1:25 Acris AP51050PU-N and 1:100 SySy 122002) and α-synuclein (1:2000 BD Biosciences 610786). For antigen retrieval, sections were treated with 99% formic acid for 3 min at room temperature. Single immunostainings of complexin 1 were performed with a peroxidase-conjugated secondary antibody and the ABC kit, and visualized with the diaminobenzidine (DAB) method. Double immunostainings with either of the complexin 1 antibodies combined with the α-synuclein antibody were performed with secondary Cy3 anti-rabbit and Alexa 488 anti-mouse antibodies. Autofluorescence was quenched with application of 0.06% Sudan Black in 70% ethanol for 10 min at room temperature. Stainings were conducted on four clinically confirmed idiopathic PD cases and two control individuals without a history of neuropsychiatric disease (PD cases 08-27, 09-12, 09-87 and 09-237; controls 12-10084 and 12-10079). The high-titer antibody detecting both complexin 1 and complexin-2 (SySy) did not stain any aggregates. Informed consent was obtained, and both the University Medical Centre Groningen and the Goethe University Hospital Frankfurt ethics commissions approved the study.

### Gene variants of *CPLX1*

Gene variants of *CPLX1* were analysed in PD and control samples that were previously described (Lavedan et al., 1998; Leroy et al., 1998; Rissling et al., 2004; Gispert et al., 2005; Möller et al., 2008) and were collected at the Düsseldorf and Marburg University Hospitals in Germany. The SNPs rs1052595, rs2242236, rs11248041, rs7340883, rs6816868 and rs10794536 were studied, using 2-5 ng genomic DNA, 1× TaqMan Genotyping Mastermix (Applied Biosystems, 4371355), 1× TaqMan SNP genotyping assay in 20 µl (Invitrogen) and running the reaction in standard conditions in a StepOnePlus apparatus (Applied Biosystems).

### Mouse breeding and characterization

Mouse breeding and characterization with brain dissection were carried out as described in the literature (Glynn et al., 2005; Gispert et al., 2009). The diet of ataxic *Cplx1*^−/−^ mice was supplemented with DietGel Recovery 72-06-5022 (ClearH_2_O). Extraction of protein and RNA was carried out as previously described (Gispert et al., 2013). The transcript expression studies used TaqMan assays (Applied Biosystems) Mm00447333_m1 (*Snca*) and Mm00446973_m1 (*Tbp*). Quantitative immunoblotting used the following primary antibodies: mouse α-synuclein (1:1000; BD Biosciences, 610786); complexin 1 (1:500; Acris, AP51050PU-N and 1:1000 SySy, 122002); β-actin (1:10,000; Sigma, A5441); and their corresponding secondary antibodies, ECL-anti-mouse-HP from sheep (GE Healthcare UK, LNA931V/AG) and ECL-anti-rabbit-HP from donkey (LNA934V/AG).

### Statistical analyses

Statistical analyses presented in bar graphs were performed using Student's unpaired *t*-tests, those of *CPLX1* level association with age or sex were performed with linear regression, runs test and two-way ANOVA, all being conducted and plotted with Prism 5.04 software (GraphPad, La Jolla, CA, USA). The allele association studies were carried out after excluding deviations from Hardy–Weinberg equilibrium by χ^2^ statistics for genotype and allele distribution.

## References

[DMM028035C1] Albers J. J., Vuletic S. and Cheung M. C. (2012). Role of plasma phospholipid transfer protein in lipid and lipoprotein metabolism. *Biochim. Biophys. Acta* 1821, 345-357. 10.1016/j.bbalip.2011.06.013 21736953 PMC3192936

[DMM028035C2] Barbour R., Kling K., Anderson J. P., Banducci K., Cole T., Diep L., Fox M., Goldstein J. M., Soriano F., Seubert P. et al. (2008). Red blood cells are the major source of alpha-synuclein in blood. *Neurodegener. Dis.* 5, 55-59. 10.1159/000112832 18182779

[DMM028035C3] Basso M., Giraudo S., Corpillo D., Bergamasco B., Lopiano L. and Fasano M. (2004). Proteome analysis of human substantia nigra in Parkinson's disease. *Proteomics* 4, 3943-3952. 10.1002/pmic.200400848 15526345

[DMM028035C4] Battisti C., Formichi P., Radi E. and Federico A. (2008). Oxidative-stress-induced apoptosis in PBLs of two patients with Parkinson disease secondary to alpha-synuclein mutation. *J. Neurol. Sci.* 267, 120-124. 10.1016/j.jns.2007.10.012 18061619

[DMM028035C5] Boeve B. F., Silber M. H., Ferman T. J., Lin S. C., Benarroch E. E., Schmeichel A. M., Ahlskog J. E., Caselli R. J., Jacobson S., Sabbagh M. et al. (2013). Clinicopathologic correlations in 172 cases of rapid eye movement sleep behavior disorder with or without a coexisting neurologic disorder. *Sleep Med.* 14, 754-762. 10.1016/j.sleep.2012.10.015 23474058 PMC3745815

[DMM028035C6] Braak H., Del Tredici K., Rüb U., de Vos R. A. I., Jansen Steur E. N. H. and Braak E. (2003). Staging of brain pathology related to sporadic Parkinson's disease. *Neurobiol. Aging* 24, 197-211. 10.1016/S0197-4580(02)00065-9 12498954

[DMM028035C7] Brehm N., Rau K., Kurz A., Gispert S. and Auburger G. (2015a). Age-related changes of 14-3-3 isoforms in midbrain of A53T-SNCA overexpressing mice. *J. Parkinsons Dis.* 5, 595-604. 10.3233/JPD-150606 26406140

[DMM028035C8] Brehm N., Bez F., Carlsson T., Kern B., Gispert S., Auburger G. and Cenci M. A. (2015b). A genetic mouse model of Parkinson's disease shows involuntary movements and increased postsynaptic sensitivity to apomorphine. *Mol. Neurobiol.* 52, 1152-1164. 10.1007/s12035-014-8911-6 25307288

[DMM028035C9] Chandra S., Fornai F., Kwon H.-B., Yazdani U., Atasoy D., Liu X., Hammer R. E., Battaglia G., German D. C., Castillo P. E. et al. (2004). Double-knockout mice for alpha- and beta-synucleins: effect on synaptic functions. *Proc. Natl. Acad. Sci. USA* 101, 14966-14971. 10.1073/pnas.0406283101 15465911 PMC522043

[DMM028035C10] Corti O., Lesage S. and Brice A. (2011). What genetics tells us about the causes and mechanisms of Parkinson's disease. *Physiol. Rev.* 91, 1161-1218. 10.1152/physrev.00022.2010 22013209

[DMM028035C11] Crabtree D., Dodson M., Ouyang X., Boyer-Guittaut M., Liang Q., Ballestas M. E., Fineberg N. and Zhang J. (2014). Over-expression of an inactive mutant cathepsin D increases endogenous alpha-synuclein and cathepsin B activity in SH-SY5Y cells. *J. Neurochem.* 128, 950-961. 10.1111/jnc.12497 24138030 PMC3951679

[DMM028035C12] Dächsel J. C., Lincoln S. J., Gonzalez J., Ross O. A., Dickson D. W. and Farrer M. J. (2007). The ups and downs of alpha-synuclein mRNA expression. *Mov. Disord.* 22, 293-295. 10.1002/mds.21223 17094104

[DMM028035C13] Diao J., Burré J., Vivona S., Cipriano D. J., Sharma M., Kyoung M., Südhof T. C. and Brunger A. T. (2013). Native alpha-synuclein induces clustering of synaptic-vesicle mimics via binding to phospholipids and synaptobrevin-2/VAMP2. *Elife* 2, e00592 10.7554/eLife.00592 23638301 PMC3639508

[DMM028035C14] Dong W., Albers J. J. and Vuletic S. (2009). Phospholipid transfer protein reduces phosphorylation of tau in human neuronal cells. *J. Neurosci. Res.* 87, 3176-3185. 10.1002/jnr.22137 19472218 PMC2755571

[DMM028035C15] Dumitriu A., Golji J., Labadorf A. T., Gao B., Beach T. G., Myers R. H., Longo K. A. and Latourelle J. C. (2016). Integrative analyses of proteomics and RNA transcriptomics implicate mitochondrial processes, protein folding pathways and GWAS loci in Parkinson disease. *BMC Med. Genomics* 9, 5 10.1186/s12920-016-0164-y 26793951 PMC4722694

[DMM028035C16] Fishbein I., Kuo Y.-M., Giasson B. I. and Nussbaum R. L. (2014). Augmentation of phenotype in a transgenic Parkinson mouse heterozygous for a Gaucher mutation. *Brain* 137, 3235-3247. 10.1093/brain/awu291 25351739 PMC4240298

[DMM028035C17] Fuchs J., Nilsson C., Kachergus J., Munz M., Larsson E.-M., Schüle B., Langston J. W., Middleton F. A., Ross O. A., Hulihan M. et al. (2007). Phenotypic variation in a large Swedish pedigree due to SNCA duplication and triplication. *Neurology* 68, 916-922. 10.1212/01.wnl.0000254458.17630.c5 17251522

[DMM028035C18] Garcia-Reitbock P., Anichtchik O., Bellucci A., Iovino M., Ballini C., Fineberg E., Ghetti B., Della Corte L., Spano P., Tofaris G. K. et al. (2010). SNARE protein redistribution and synaptic failure in a transgenic mouse model of Parkinson's disease. *Brain* 133, 2032-2044. 10.1093/brain/awq132 20534649 PMC2892942

[DMM028035C19] Gardai S. J., Mao W., Schüle B., Babcock M., Schoebel S., Lorenzana C., Alexander J., Kim S., Glick H., Hilton K. et al. (2013). Elevated alpha-synuclein impairs innate immune cell function and provides a potential peripheral biomarker for Parkinson's disease. *PLoS ONE* 8, e71634 10.1371/journal.pone.0071634 24058406 PMC3751933

[DMM028035C20] Gautier T. and Lagrost L. (2011). Plasma PLTP (phospholipid-transfer protein): an emerging role in ‘reverse lipopolysaccharide transport’ and innate immunity. *Biochem. Soc. Trans.* 39, 984-988. 10.1042/BST0390984 21787334

[DMM028035C21] Gispert S., Trenkwalder C., Mota-Vieira L., Kostic V. and Auburger G. (2005). Failure to find alpha-synuclein gene dosage changes in 190 patients with familial Parkinson disease. *Arch. Neurol.* 62, 96-98. 10.1001/archneur.62.1.96 15642855

[DMM028035C22] Gispert S., Ricciardi F., Kurz A., Azizov M., Hoepken H.-H., Becker D., Voos W., Leuner K., Müller W. E., Kudin A. P. et al. (2009). Parkinson phenotype in aged PINK1-deficient mice is accompanied by progressive mitochondrial dysfunction in absence of neurodegeneration. *PLoS ONE* 4, e5777 10.1371/journal.pone.0005777 19492057 PMC2686165

[DMM028035C23] Gispert S., Parganlija D., Klinkenberg M., Drose S., Wittig I., Mittelbronn M., Grzmil P., Koob S., Hamann A., Walter M. et al. (2013). Loss of mitochondrial peptidase Clpp leads to infertility, hearing loss plus growth retardation via accumulation of CLPX, mtDNA and inflammatory factors. *Hum. Mol. Genet.* 22, 4871-4887. 10.1093/hmg/ddt338 23851121 PMC7108587

[DMM028035C24] Gispert S., Kurz A., Brehm N., Rau K., Walter M., Riess O. and Auburger G. (2015a). Complexin-1 and Foxp1 expression changes are novel brain effects of alpha-synuclein pathology. *Mol. Neurobiol.* 52, 57-63. 10.1007/s12035-014-8844-0 25112678 PMC4510914

[DMM028035C25] Gispert S., Brehm N., Weil J., Seidel K., Rub U., Kern B., Walter M., Roeper J. and Auburger G. (2015b). Potentiation of neurotoxicity in double-mutant mice with Pink1 ablation and A53T-SNCA overexpression. *Hum. Mol. Genet.* 24, 1061-1076. 10.1093/hmg/ddu520 25296918 PMC4986551

[DMM028035C26] Glynn D., Drew C. J., Reim K., Brose N. and Morton A. J. (2005). Profound ataxia in complexin I knockout mice masks a complex phenotype that includes exploratory and habituation deficits. *Hum. Mol. Genet.* 14, 2369-2385. 10.1093/hmg/ddi239 16000319

[DMM028035C27] Goedert M., Spillantini M. G., Del Tredici K. and Braak H. (2013). 100 years of Lewy pathology. *Nat. Rev. Neurol.* 9, 13-24. 10.1038/nrneurol.2012.242 23183883

[DMM028035C28] Golbe L. I., Di Iorio G., Bonavita V., Miller D. C. and Duvoisin R. C. (1990). A large kindred with autosomal dominant Parkinson's disease. *Ann. Neurol.* 27, 276-282. 10.1002/ana.410270309 2158268

[DMM028035C29] Hughes A. J., Daniel S. E., Kilford L. and Lees A. J. (1992). Accuracy of clinical diagnosis of idiopathic Parkinson's disease: a clinico-pathological study of 100 cases. *J. Neurol. Neurosurg. Psychiatry* 55, 181-184. 10.1136/jnnp.55.3.181 1564476 PMC1014720

[DMM028035C30] Iczkiewicz J., Jackson M. J., Smith L. A., Rose S. and Jenner P. (2006). Osteopontin expression in substantia nigra in MPTP-treated primates and in Parkinson's disease. *Brain Res.* 1118, 239-250. 10.1016/j.brainres.2006.08.036 16962083

[DMM028035C31] Iranzo A., Tolosa E., Gelpi E., Molinuevo J. L., Valldeoriola F., Serradell M., Sanchez-Valle R., Vilaseca I., Lomeña F., Vilas D. et al. (2013). Neurodegenerative disease status and post-mortem pathology in idiopathic rapid-eye-movement sleep behaviour disorder: an observational cohort study. *Lancet Neurol.* 12, 443-453. 10.1016/S1474-4422(13)70056-5 23562390

[DMM028035C32] Iranzo A., Fernández-Arcos A., Tolosa E., Serradell M., Molinuevo J. L., Valldeoriola F., Gelpi E., Vilaseca I., Sánchez-Valle R., Lladó A. et al. (2014). Neurodegenerative disorder risk in idiopathic REM sleep behavior disorder: study in 174 patients. *PLoS ONE* 9, e89741 10.1371/journal.pone.0089741 24587002 PMC3935943

[DMM028035C33] Janezic S., Threlfell S., Dodson P. D., Dowie M. J., Taylor T. N., Potgieter D., Parkkinen L., Senior S. L., Anwar S., Ryan B. et al. (2013). Deficits in dopaminergic transmission precede neuron loss and dysfunction in a new Parkinson model. *Proc. Natl. Acad. Sci. USA* 110, E4016-E4025. 10.1073/pnas.1309143110 24082145 PMC3801069

[DMM028035C34] Kim S., Jeon B. S., Heo C., Im P. S., Ahn T. B., Seo J. H., Kim H. S., Park C. H., Choi S. H., Cho S. H. et al. (2004). Alpha-synuclein induces apoptosis by altered expression in human peripheral lymphocyte in Parkinson's disease. *FASEB J.* 18, 1615-1617. 10.1096/fj.04-1917fje 15289452

[DMM028035C35] Kurz A., Double K. L., Lastres-Becker I., Tozzi A., Tantucci M., Bockhart V., Bonin M., García-Arencibia M., Nuber S., Schlaudraff F. et al. (2010). A53T-alpha-synuclein overexpression impairs dopamine signaling and striatal synaptic plasticity in old mice. *PLoS ONE* 5, e11464 10.1371/journal.pone.0011464 20628651 PMC2898885

[DMM028035C36] Lavedan C.,, Buchholtz S., Auburger G., Albin R. L., Athanassiadou A., Blancato J., Burguera J. A., Ferrell R. E., Kostic V., Leroy E. et al. (1998). Absence of mutation in the beta- and gamma-synuclein genes in familial autosomal dominant Parkinson's disease. *DNA Res.* 5, 401-402. 10.1093/dnares/5.6.401 10048491

[DMM028035C37] Leroy E., Boyer R., Auburger G., Leube B., Ulm G., Mezey E., Harta G., Brownstein M. J., Jonnalagada S., Chernova T. et al. (1998). The ubiquitin pathway in Parkinson's disease. *Nature* 395, 451-452. 10.1038/26652 9774100

[DMM028035C38] Lill C. M., Roehr J. T., McQueen M. B., Kavvoura F. K., Bagade S., Schjeide B.-M. M., Schjeide L. M., Meissner E., Zauft U., Allen N. C. et al. (2012). Comprehensive research synopsis and systematic meta-analyses in Parkinson's disease genetics: the PDGene database. *PLoS Genet.* 8, e1002548 10.1371/journal.pgen.1002548 22438815 PMC3305333

[DMM028035C39] Livak K. J. and Schmittgen T. D. (2001). Analysis of relative gene expression data using real-time quantitative PCR and the 2(−Delta Delta C(T)). Method. *Methods* 25, 402-408. 10.1006/meth.2001.1262 11846609

[DMM028035C40] Maetzler W., Berg D., Schalamberidze N., Melms A., Schott K., Mueller J. C., Liaw L., Gasser T. and Nitsch C. (2007). Osteopontin is elevated in Parkinson's disease and its absence leads to reduced neurodegeneration in the MPTP model. *Neurobiol. Dis.* 25, 473-482. 10.1016/j.nbd.2006.10.020 17188882

[DMM028035C41] Mahowald M. W. and Schenck C. H. (2013). REM sleep behaviour disorder: a marker of synucleinopathy. *Lancet Neurol.* 12, 417-419. 10.1016/S1474-4422(13)70078-4 23578776

[DMM028035C42] Marek K., Jennings D., Lasch S., Siderowf A., Tanner C., Simuni T., Coffey C., Kieburtz K., Flagg E., Chowdhury S. et al. (2011). The Parkinson Progression Marker Initiative (PPMI). *Prog. Neurobiol.* 95, 629-635. 10.1016/j.pneurobio.2011.09.005 21930184 PMC9014725

[DMM028035C43] Möller J. C., Rissling I., Mylius V., Höft C., Eggert K. M. and Oertel W. H. (2008). The prevalence of the G2019S and R1441C/G/H mutations in LRRK2 in German patients with Parkinson's disease. *Eur. J. Neurol.* 15, 743-745. 10.1111/j.1468-1331.2008.02154.x 18484993

[DMM028035C44] Mortazavi A., Williams B. A., McCue K., Schaeffer L. and Wold B. (2008). Mapping and quantifying mammalian transcriptomes by RNA-Seq. *Nat. Methods* 5, 621-628. 10.1038/nmeth.1226 18516045 PMC13303166

[DMM028035C45] Mukaetova-Ladinska E. B., Andras A., Milne J., Abdel-All Z., Borr I., Jaros E., Perry R. H., Honer W. G., Cleghorn A., Doherty J. et al. (2013). Synaptic proteins and choline acetyltransferase loss in visual cortex in dementia with Lewy bodies. *J. Neuropathol. Exp. Neurol.* 72, 53-60. 10.1097/NEN.0b013e31827c5710 23242284

[DMM028035C46] Mutez E., Leprêtre F., Le Rhun E., Larvor L., Duflot A., Mouroux V., Kerckaert J.-P., Figeac M., Dujardin K., Destée A. et al. (2011). SNCA locus duplication carriers: from genetics to Parkinson disease phenotypes. *Hum. Mutat.* 32, E2079-E2090. 10.1002/humu.21459 21412942

[DMM028035C47] Nakata Y., Yasuda T., Fukaya M., Yamamori S., Itakura M., Nihira T., Hayakawa H., Kawanami A., Kataoka M., Nagai M. et al. (2012). Accumulation of alpha-synuclein triggered by presynaptic dysfunction. *J. Neurosci.* 32, 17186-17196. 10.1523/JNEUROSCI.2220-12.2012 23197711 PMC6621870

[DMM028035C48] Nalls M. A., Pankratz N., Lill C. M., Do C. B., Hernandez D. G., Saad M., DeStefano A. L., Kara E., Bras J., Sharma M. et al. (2014). Large-scale meta-analysis of genome-wide association data identifies six new risk loci for Parkinson's disease. *Nat. Genet.* 46, 989-993. 10.1038/ng.3043 25064009 PMC4146673

[DMM028035C49] Nemani V. M., Lu W., Berge V., Nakamura K., Onoa B., Lee M. K., Chaudhry F. A., Nicoll R. A. and Edwards R. H. (2010). Increased expression of alpha-synuclein reduces neurotransmitter release by inhibiting synaptic vesicle reclustering after endocytosis. *Neuron* 65, 66-79. 10.1016/j.neuron.2009.12.023 20152114 PMC3119527

[DMM028035C50] Nishioka K., Hayashi S., Farrer M. J., Singleton A. B., Yoshino H., Imai H., Kitami T., Sato K., Kuroda R., Tomiyama H. et al. (2006). Clinical heterogeneity of alpha-synuclein gene duplication in Parkinson's disease. *Ann. Neurol.* 59, 298-309. 10.1002/ana.20753 16358335

[DMM028035C51] Park S. M., Jung H. Y., Kim H. O., Rhim H., Paik S. R., Chung K. C., Park J. H. and Kim J. (2002). Evidence that alpha-synuclein functions as a negative regulator of Ca(++)-dependent alpha-granule release from human platelets. *Blood* 100, 2506-2514. 10.1182/blood.V100.7.2506 12239163

[DMM028035C52] Piccoli G., Condliffe S. B., Bauer M., Giesert F., Boldt K., De Astis S., Meixner A., Sarioglu H., Vogt-Weisenhorn D. M., Wurst W. et al. (2011). LRRK2 controls synaptic vesicle storage and mobilization within the recycling pool. *J. Neurosci.* 31, 2225-2237. 10.1523/JNEUROSCI.3730-10.2011 21307259 PMC6633036

[DMM028035C53] Platt N. J., Gispert S., Auburger G. and Cragg S. J. (2012). Striatal dopamine transmission is subtly modified in human A53Talpha-synuclein overexpressing mice. *PLoS ONE* 7, e36397 10.1371/journal.pone.0036397 22570709 PMC3343082

[DMM028035C54] Polymeropoulos M. H., Higgins J. J., Golbe L. I., Johnson W. G., Ide S. E., Di Iorio G., Sanges G., Stenroos E. S., Pho L. T., Schaffer A. A. et al. (1996). Mapping of a gene for Parkinson's disease to chromosome 4q21-q23. *Science* 274, 1197-1199. 10.1126/science.274.5290.1197 8895469

[DMM028035C55] Polymeropoulos M. H., Lavedan C., Leroy E., Ide S. E., Dehejia A., Dutra A., Pike B., Root H., Rubenstein J., Boyer R. et al. (1997). Mutation in the alpha-synuclein gene identified in families with Parkinson's disease. *Science* 276, 2045-2047. 10.1126/science.276.5321.2045 9197268

[DMM028035C56] Postuma R. B., Aarsland D., Barone P., Burn D. J., Hawkes C. H., Oertel W. and Ziemssen T. (2012). Identifying prodromal Parkinson's disease: pre-motor disorders in Parkinson's disease. *Mov. Disord.* 27, 617-626. 10.1002/mds.24996 22508280

[DMM028035C57] Reheman A., Tasneem S., Ni H. and Hayward C. P. M. (2010). Mice with deleted multimerin 1 and alpha-synuclein genes have impaired platelet adhesion and impaired thrombus formation that is corrected by multimerin 1. *Thromb. Res.* 125, e177-e183. 10.1016/j.thromres.2010.01.009 20138333

[DMM028035C58] Reim K., Mansour M., Varoqueaux F., McMahon H. T., Südhof T. C., Brose N. and Rosenmund C. (2001). Complexins regulate a late step in Ca2+-dependent neurotransmitter release. *Cell* 104, 71-81. 10.1016/S0092-8674(01)00192-1 11163241

[DMM028035C59] Rhinn H., Qiang L., Yamashita T., Rhee D., Zolin A., Vanti W. and Abeliovich A. (2012). Alternative alpha-synuclein transcript usage as a convergent mechanism in Parkinson's disease pathology. *Nat. Commun.* 3, 1084 10.1038/ncomms2032 23011138 PMC3660047

[DMM028035C60] Rissling I., Geller F., Bandmann O., Stiasny-Kolster K., Körner Y., Meindorfner C., Krüger H.-P., Oertel W. H. and Möller J. C. (2004). Dopamine receptor gene polymorphisms in Parkinson's disease patients reporting “sleep attacks”. *Mov. Disord.* 19, 1279-1284. 10.1002/mds.20245 15390060

[DMM028035C61] Robinson M. D., McCarthy D. J. and Smyth G. K. (2010). edgeR: a Bioconductor package for differential expression analysis of digital gene expression data. *Bioinformatics* 26, 139-140. 10.1093/bioinformatics/btp616 19910308 PMC2796818

[DMM028035C62] Santiago J. A. and Potashkin J. A. (2015). Network-based metaanalysis identifies HNF4A and PTBP1 as longitudinally dynamic biomarkers for Parkinson's disease. *Proc. Natl. Acad. Sci. USA* 112, 2257-2262. 10.1073/pnas.1423573112 25646437 PMC4343174

[DMM028035C63] Scherzer C. R., Eklund A. C., Morse L. J., Liao Z., Locascio J. J., Fefer D., Schwarzschild M. A., Schlossmacher M. G., Hauser M. A., Vance J. M. et al. (2007). Molecular markers of early Parkinson's disease based on gene expression in blood. *Proc. Natl. Acad. Sci. USA* 104, 955-960. 10.1073/pnas.0610204104 17215369 PMC1766335

[DMM028035C64] Seidel K., den Dunnen W. F. A., Schultz C., Paulson H., Frank S., de Vos R. A., Brunt E. R., Deller T., Kampinga H. H. and Rüb U. (2010a). Axonal inclusions in spinocerebellar ataxia type 3. *Acta Neuropathol.* 120, 449-460. 10.1007/s00401-010-0717-7 20635090 PMC2923324

[DMM028035C65] Seidel K., Schöls L., Nuber S., Petrasch-Parwez E., Gierga K., Wszolek Z., Dickson D., Gai W. P., Bornemann A., Riess O. et al. (2010b). First appraisal of brain pathology owing to A30P mutant alpha-synuclein. *Ann. Neurol.* 67, 684-689. 10.1002/ana.22078 20437567

[DMM028035C66] Sharon R., Bar-Joseph I., Frosch M. P., Walsh D. M., Hamilton J. A. and Selkoe D. J. (2003). The formation of highly soluble oligomers of alpha-synuclein is regulated by fatty acids and enhanced in Parkinson's disease. *Neuron* 37, 583-595. 10.1016/S0896-6273(03)00024-2 12597857

[DMM028035C67] Shi M., Movius J., Dator R., Aro P., Zhao Y., Pan C., Lin X., Bammler T. K., Stewart T., Zabetian C. P. et al. (2015). Cerebrospinal fluid peptides as potential Parkinson disease biomarkers: a staged pipeline for discovery and validation. *Mol. Cell. Proteomics* 14, 544-555. 10.1074/mcp.m114.040576 25556233 PMC4349976

[DMM028035C68] Shin E. C., Cho S. E., Lee D.-K., Hur M.-W., Paik S. R., Park J. H. and Kim J. (2000). Expression patterns of alpha-synuclein in human hematopoietic cells and in Drosophila at different developmental stages. *Mol. Cells* 10, 65-70. 10.1007/s10059-000-0065-x 10774749

[DMM028035C69] Sidransky E. and Lopez G. (2012). The link between the GBA gene and parkinsonism. *Lancet Neurol.* 11, 986-998. 10.1016/S1474-4422(12)70190-4 23079555 PMC4141416

[DMM028035C70] Singleton A. B., Farrer M., Johnson J., Singleton A., Hague S., Kachergus J., Hulihan M., Peuralinna T., Dutra A., Nussbaum R. et al. (2003). alpha-Synuclein locus triplication causes Parkinson's disease. *Science* 302, 841 10.1126/science.1090278 14593171

[DMM028035C71] Stiasny-Kolster K., Doerr Y., Moller J. C., Hoffken H., Behr T. M., Oertel W. H. and Mayer G. (2005). Combination of ‘idiopathic’ REM sleep behaviour disorder and olfactory dysfunction as possible indicator for alpha-synucleinopathy demonstrated by dopamine transporter FP-CIT-SPECT. *Brain* 128, 126-137. 10.1093/brain/awh322 15548552

[DMM028035C72] Subramanian A., Tamayo P., Mootha V. K., Mukherjee S., Ebert B. L., Gillette M. A., Paulovich A., Pomeroy S. L., Golub T. R., Lander E. S. et al. (2005). Gene set enrichment analysis: a knowledge-based approach for interpreting genome-wide expression profiles. *Proc. Natl. Acad. Sci. USA* 102, 15545-15550. 10.1073/pnas.0506580102 16199517 PMC1239896

[DMM028035C73] Subramanian A., Kuehn H., Gould J., Tamayo P. and Mesirov J. P. (2007). GSEA-P: a desktop application for Gene Set Enrichment Analysis. *Bioinformatics* 23, 3251-3253. 10.1093/bioinformatics/btm369 17644558

[DMM028035C74] Subramaniam M., Althof D., Gispert S., Schwenk J., Auburger G., Kulik A., Fakler B. and Roeper J. (2014). Mutant alpha-synuclein enhances firing frequencies in dopamine substantia nigra neurons by oxidative impairment of A-type potassium channels. *J. Neurosci.* 34, 13586-13599. 10.1523/JNEUROSCI.5069-13.2014 25297088 PMC6608377

[DMM028035C75] Tozzi A., Costa C., Siliquini S., Tantucci M., Picconi B., Kurz A., Gispert S., Auburger G. and Calabresi P. (2012). Mechanisms underlying altered striatal synaptic plasticity in old A53T-alpha synuclein overexpressing mice. *Neurobiol. Aging* 33, 1792-1799. 10.1016/j.neurobiolaging.2011.05.002 21684039

[DMM028035C76] Tsujimura A., Taguchi K., Watanabe Y., Tatebe H., Tokuda T., Mizuno T. and Tanaka M. (2014). Lysosomal enzyme cathepsin B enhances the aggregate forming activity of exogenous alpha-synuclein fibrils. *Neurobiol. Dis.* 73C, 244-253. 10.1016/j.nbd.2014.10.011 25466281

[DMM028035C77] Westbroek W., Gustafson A. M. and Sidransky E. (2011). Exploring the link between glucocerebrosidase mutations and parkinsonism. *Trends Mol. Med.* 17, 485-493. 10.1016/j.molmed.2011.05.003 21723784 PMC3351003

[DMM028035C78] Yap T. L., Gruschus J. M., Velayati A., Westbroek W., Goldin E., Moaven N., Sidransky E. and Lee J. C. (2011). Alpha-synuclein interacts with Glucocerebrosidase providing a molecular link between Parkinson and Gaucher diseases. *J. Biol. Chem.* 286, 28080-28088. 10.1074/jbc.M111.237859 21653695 PMC3151053

[DMM028035C79] Yasuda T., Nakata Y., Choong C.-J. and Mochizuki H. (2013). Neurodegenerative changes initiated by presynaptic dysfunction. *Transl. Neurodegener.* 2, 16 10.1186/2047-9158-2-16 23919415 PMC3750287

